# Experimental and Theoretical Evaluation of Notch Strength in MEX-Printed Acrylonitrile Butadiene Styrene Under Symmetrical Three-Point Bending

**DOI:** 10.3390/polym18212799

**Published:** 2026-11-09

**Authors:** Ali Reza Torabi, Media Naeimayi, Andres Garcia Granada, Majid Reza Ayatollahi

**Affiliations:** 1 Fracture Research Laboratory, College of Interdisciplinary Science and Technology, University of Tehran, Tehran 1439957131, Iran; 2 Fatigue and Fracture Research Laboratory, Center of Excellence in Experimental Solid Mechanics and Dynamics, School of Mechanical Engineering, Iran University of Science and Technology, Narmak, Tehran 1684613114, Iranm.ayat@iust.ac.ir (M.R.A.); 3 Grup d’Enginyeria en Producte Industrial (GEPI), Institut Químic de Sarrià, Universitat Ramon Llull, E08017 Barcelona, Spain

**Keywords:** fracture, notch, FDM-printing, acrylonitrile butadiene styrene (ABS), symmetrical three-point bending, Virtual Isotropic Material Concept (VIMC)

## Abstract

Material extrusion (MEX) is an extensively used additive manufacturing technique that facilitates the fabrication of precise physical components with complex geometries through successive material deposition. However, the anisotropic behavior inherent to MEX-printed materials can substantially influence their fracture response. This study examines the fracture behavior of U- and blunt V-notched (BV-notched) MEX-printed Acrylonitrile Butadiene Styrene (ABS) under symmetrical three-point bending using rectangular specimens with a symmetrical [0/90]_s_ cross-ply printing architecture. To this end, three different notch root radii within the 1–4 mm range are experimentally investigated, and the measured fracture loads are compared with theoretical-numerical predictions obtained by integrating the virtual isotropic material concept (VIMC) with four failure models, namely the point stress (PS), mean stress (MS), and averaged strain energy density (ASED) criteria, as well as the extended finite element method coupled with a cohesive zone model (XFEM-CZM). All four VIMC-based criteria provide reasonable correspondence with the experimental results. Among them, the ASED-VIMC criterion exhibits the lowest average percentage difference of 5.7% for the 1–4 mm range of notch root radius, symmetrical [0/90]_s_ cross-ply printing architecture, and symmetrical three-point bending, followed by the PS-VIMC criterion with 7.4%. ASED-VIMC criterion also demonstrates the most consistent performance for both notch configurations, likely due to its lower sensitivity to notch geometry.

## 1. Introduction

Additive manufacturing (AM) fabricates components through the controlled, layer-by-layer deposition of material on a temperature-controlled platform, guided by a computer-driven nozzle capable of motion in three-dimensional space. These features provide opportunities for producing geometrically intricate components with high precision, while obviating the necessity for conventional machining, cutting, and molding processes. AM technology encompasses seven distinct process categories, namely powder bed fusion (PBF), directed energy deposition (DED), binder jetting (BJT), material extrusion (MEX), material jetting (MJT), vat photopolymerization (VPP), and sheet lamination (SHL) [1]. Among these, material extrusion (MEX), commonly known as fused deposition modeling (FDM) technology, is one of the most accessible and widely studied additive manufacturing processes for structural polymer components, owing to its compatibility with many common thermoplastics, such as acrylonitrile butadiene styrene (ABS), polylactic acid (PLA), polyphenysulfone (PPSU), polyethyleneterephthalate (PET), and polyamide (PA) [2,3].

Characterizing the mechanical and fracture behavior of MEX-printed parts is strongly governed by the selected printing parameters, including the layer thickness, printing speed, and printing architecture, all of which influence interlayer bond quality and the resulting mechanical anisotropy [4,5]. Christiyan et al. [6] showed that lower layer thicknesses and printing speeds can improve the tensile and flexural strengths in ABS composites. This finding was supported by Agarwal et al. [7], who identified the layer thickness and printing speed as the most significant parameters affecting dimensional accuracy and repeatability. Dusanapudi et al. [8] combined grey relational analysis with a genetic algorithm to show that an extrusion multiplier of 1, a temperature of 240 °C, and a 0.1 mm layer thickness significantly enhance mechanical properties while reducing porosity. Furthermore, Popescu et al. [9] emphasized that the combined influence of parameters is mandatory to ensure that printed objects meet structural requirements.

In addition to the manufacturing parameters, the presence of a notch with finite root radius can have a significant influence on the fracture behavior of polymer components. A finite-radius notch generally increases the apparent fracture resistance compared with a sharp crack of the same depth [10]. Consequently, treating a notch as a crack often leads to conservative fracture assessments, a phenomenon commonly referred to as the notch effect. The severity of this effect depends on both the notch geometry and the material characteristics [10]. Moreover, notches are intentional defects that introduce stress concentrations but do not necessarily lead to immediate failure. Rather, caution is essential in the deliberate use of notches, as they act as preferential sites for the initiation and propagation of cracks. Accordingly, accounting for their influence is essential for reliable fracture assessment and integrity in structural designs. Cicero et al. extended the theory of critical distances (TCD) across five additively manufactured materials, including ABS, PLA, acrylonitrile-styrene-acrylate (ASA), graphene-reinforced PLA (PLA-Gr) and carbon-fiber-reinforced ASA (ASA-CF) [11,12,13,14]. They reported that PLA typically exhibits stronger notch sensitivity than ASA, showing the lowest fracture toughness value. The two aforementioned composites revealed higher fracture toughness than the pristine polymers; however, their notch sensitivity did not follow a consistent trend [10].

The printing architecture is among the primary sources of anisotropic behavior in MEX-printed parts; therefore, characterizing the fracture response under varying architectures is essential for predicting material limitations and designing reliable, well-optimized structures [15]. The layer-by-layer fabrication process leads to inherent dependency of material on the orientation of deposited layers, as well as the quality of interlayer bonding [16]. Zhang et al. [17] found that additively manufactured PLA, ABS, and PA consistently demonstrate anisotropic fracture toughness across three different printing architectures, with the lowest fracture toughness value occurring exclusively in the 90° transverse printing direction. Similarly, Sotia et al. [18] highlighted pronounced anisotropy in the mode I fracture toughness in single-edge notched bend samples produced by selective laser sintering (SLS), with a 0° build orientation consistently yielding the highest density and fracture toughness. To account for material anisotropy in a bulk manner rather than relying on layer-by-layer characterization, Torabi and Pirhadi [19,20,21] introduced the virtual isotropic material concept (VIMC) for fiber-reinforced polymer composites. Torabi et al. and Ghanbari et al. [22,23] linked the VIMC to the mean stress (MS), point stress (PS), and averaged strain energy density (ASED) criteria for fracture prediction of U- and blunt V-notched ABS, respectively, and in both cases found that the MS criterion provides the most accurate predictions. Alirezaeipour et al. [24] further extended the framework by coupling VIMC with the finite fracture mechanics (FFM) to predict the last-ply failure in keyhole-notched laminates, demonstrating its applicability through a practical and low-cost approach. In a related study, BV-notched diagonally loaded square-plate PLA specimens were assessed using VIMC combined with the modified equivalent material concept (MEMC), together with the maximum tangential stress (MTS) and MS criteria. It was revealed that both criteria provide accurate predictions of fracture loads, indicating that the predictions were not considerably sensitive to the notch shape [25].

Finding how 3D-printed materials respond under varying stresses and loading conditions is essential for predicting failure in functional components. Notched MEX parts have been widely examined for fracture under different loading conditions, as addressed in the following studies. Vălean et al. [26] used symmetric and asymmetric four-point bending on single-edge-notched bend specimens to determine Mode I and Mode II fracture toughness values, respectively, finding that printed notches yield higher fracture resistance and lower experimental dispersion than milled notches. This superior performance was attributed to the higher geometrical accuracy of the printed notch and preservation of structural integrity when the notch geometry was printed directly rather than machined. Vălean et al. [27] subsequently demonstrated that introducing a notch under three-point bending significantly reduces mechanical performance and maximum flexural strength for virgin PLA. For unidirectional MEX-printed ABS specifically, Shahbaz et al. [28] showed that crack initiation is not affected by material nature, particularly isotropic or anisotropic, owing to the symmetry in geometry and loading conditions. Soltanmohammadi et al. [29] investigated the fracture behavior of ABS–TPU blends fabricated using material extrusion under tensile, compression, and bending tests. They reported that increasing the TPU content lowers the crack-initiation force and shifts the behavior from brittle towards ductile. Bending tests have also been employed to assess the mechanical behavior of different additively manufactured materials [30], including carbon fiber reinforced polymer (CFRP) composites with Bouligand meso-structure [31], Polyethylene Terephthalate Glycol (PETG) [32], polyamide [18,33], continuous carbon fiber reinforced composites (CCFRCs) [34,35] and continuous carbon-fiber-reinforced PLA (CCF/PLA) [36].

Nearly all of the evidence reviewed above has provided valuable insights into the effects of printing architecture, notch geometry, and loading condition on the fracture behavior of MEX-printed materials. However, the incorporation of both U- and BV-notches within a single symmetrical [0/90]_s_ cross-ply MEX-printed ABS specimen tested under a bending-induced stress gradient rather than uniform tension remains unclear. Accordingly, this study assesses the fracture behavior of rectangular three-point-bend (TPB) specimens of MEX-printed ABS with a symmetrical [0/90]_s_ printing architecture, containing single-edge U- and BV-notches across three notch root radii. Fracture loads are measured experimentally and compared with theoretical predictions obtained by linking VIMC to the XFEM-CZM model, together with the MS, PS, and ASED criteria. Particular attention is paid to the influence of notch profile and root radius on the fracture response and to the applicability of the different prediction approaches under bending-dominated loading. The results provide further insight into the combined influence of notch geometry and loading configuration on the fracture assessment of MEX-printed ABS components.

## 2. Materials and Methods

This section describes the material and specimen fabrication procedures, followed by the experimental setup used for the characterization of MEX-printed ABS TPB specimens with a symmetrical [0/90]_s_ printing architecture.

### 2.1. Material Identification

All the specimens are printed from ABS filament supplied by YOUSU, a Chinese producer, with a nominal diameter and density of 1.75 mm and 1.05 g/cm^3^, respectively. ABS exhibits favorable mechanical and physical properties as a popular material used in extrusion-based 3D printing. Compared with other printable thermoplastic materials, ABS offers superior toughness, flexibility, and impact resistance. Furthermore, it can withstand higher operational temperatures without deforming, owing to its higher glass transition temperature (~105 °C) relative to PLA, thereby resulting in improved dimensional stability [37,38].

Since the printing parameters adopted in the present study are identical to those employed in our previous investigation on tensile specimens of the same material and layup [39], the main mechanical properties of the cross-ply ABS are taken from the previous work and listed in Table 1. The ABS filaments used in both investigations were purchased together from the same manufacturer (YOUSU), and all specimens were fabricated using the same 3D printer. Therefore, additional tensile tests were not performed in the present study. Note that the values in the parentheses represent the standard deviation.

### 2.2. Preparation of Samples

**Figure 1:**
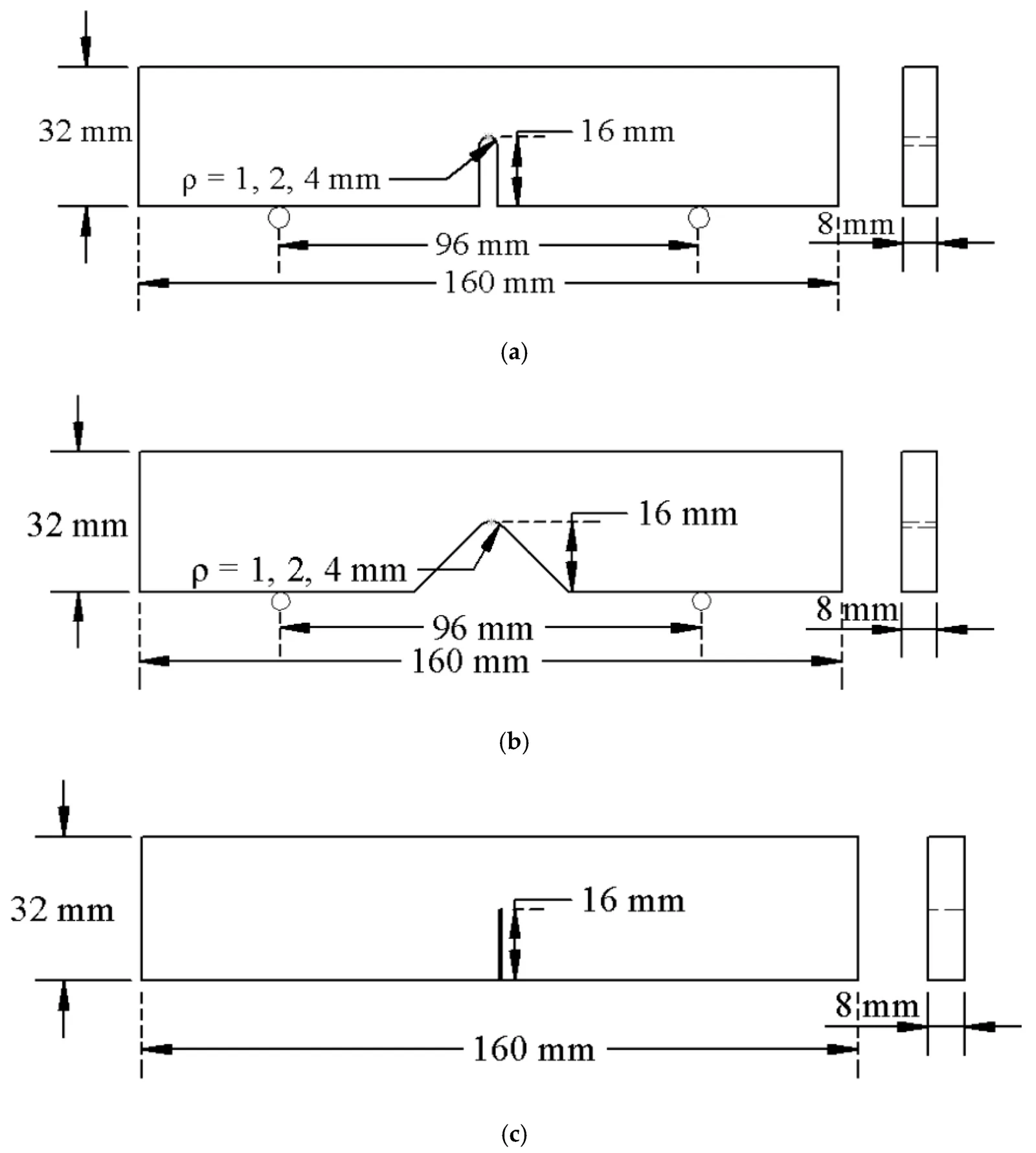
Schematic drawing of (**a**) U-notched, (**b**) BV-notched, and (**c**) pre-cracked TPB samples.

Rectangular three-point-bend (TPB) specimens containing a single-edge notch are fabricated across the U- and BV-notch geometries, and three distinct notch root radii are examined. The notches are produced directly during the printing process as part of the deposited layers. Consequently, possible additional residual stresses associated with post-print machining of notches in MEX-printed parts are minimized. Separately, pre-cracked TPB specimens are used for apparent fracture toughness characterization. Therefore, a 16 mm long crack is introduced into the samples at the same location as the notches. 15.5 mm out of 16 mm is produced directly by the printer, while the remaining 0.5 mm is generated by hand using a razor blade to sharpen the crack tip. Though the same procedure is used to sharpen the crack tip in the three pre-cracked specimens with a razor blade, some variations in the crack tip radius may exist, as the additional 0.5 mm crack is introduced by hand operation. The exact crack-tip radius is not independently measured herein, and therefore its geometrical consistency cannot be quantitatively verified. The geometry of the TPB specimens is shown in Figure 1, where ρ denotes the notch root radius.

The mechanical properties of printed samples are greatly affected by the manufacturing process parameters, including the nozzle diameter, layer height (the thickness of each deposited layer), extrusion width (the width of extruded thermoplastic material, or the center-to-center distance between adjacent deposited single-line material, named road), nozzle temperature, bed temperature, infill percentage, printing architecture (e.g., [0/90]_s_), and printing speed. These parameters collectively contribute to the desired mechanical properties of the printed part. The selection of printing parameters involves a comprehensive series of preliminary examinations to determine suitable processing conditions while diminishing printing-related defects. A constant set of process parameters is maintained for all specimens. The applied process parameters are listed in Table 2. All specimens are printed flat on the printing bed. Considering the specimen thickness of 8 mm and the layer height of 0.25 mm, each specimen comprises 32 deposited layers. The 0° and 90° raster orientations are defined as perpendicular and parallel to the applied loading direction, respectively. To obtain a symmetrical cross-ply printing architecture, the raster orientations are alternated between 0° and 90° in the first 16 layers, followed by their mirrored sequence with respect to the mid-thickness in the remaining 16 layers.

### 2.3. Test Procedure and Results

As reported in [40], moisture absorption has a significant adverse influence on the mechanical properties of 3D-printed polymers. Therefore, appropriate storage conditions are employed to minimize moisture absorption and improve the reliability of the experimental results, while reducing the data scatter. Accordingly, MEX-printed samples are preserved in vacuum-sealed plastic bags containing silica gel. Furthermore, each MEX-printed sample is printed at the center of the printing bed, leading to reduced position-dependent variability and dispersion in the measured mechanical properties.

**Figure 2:**
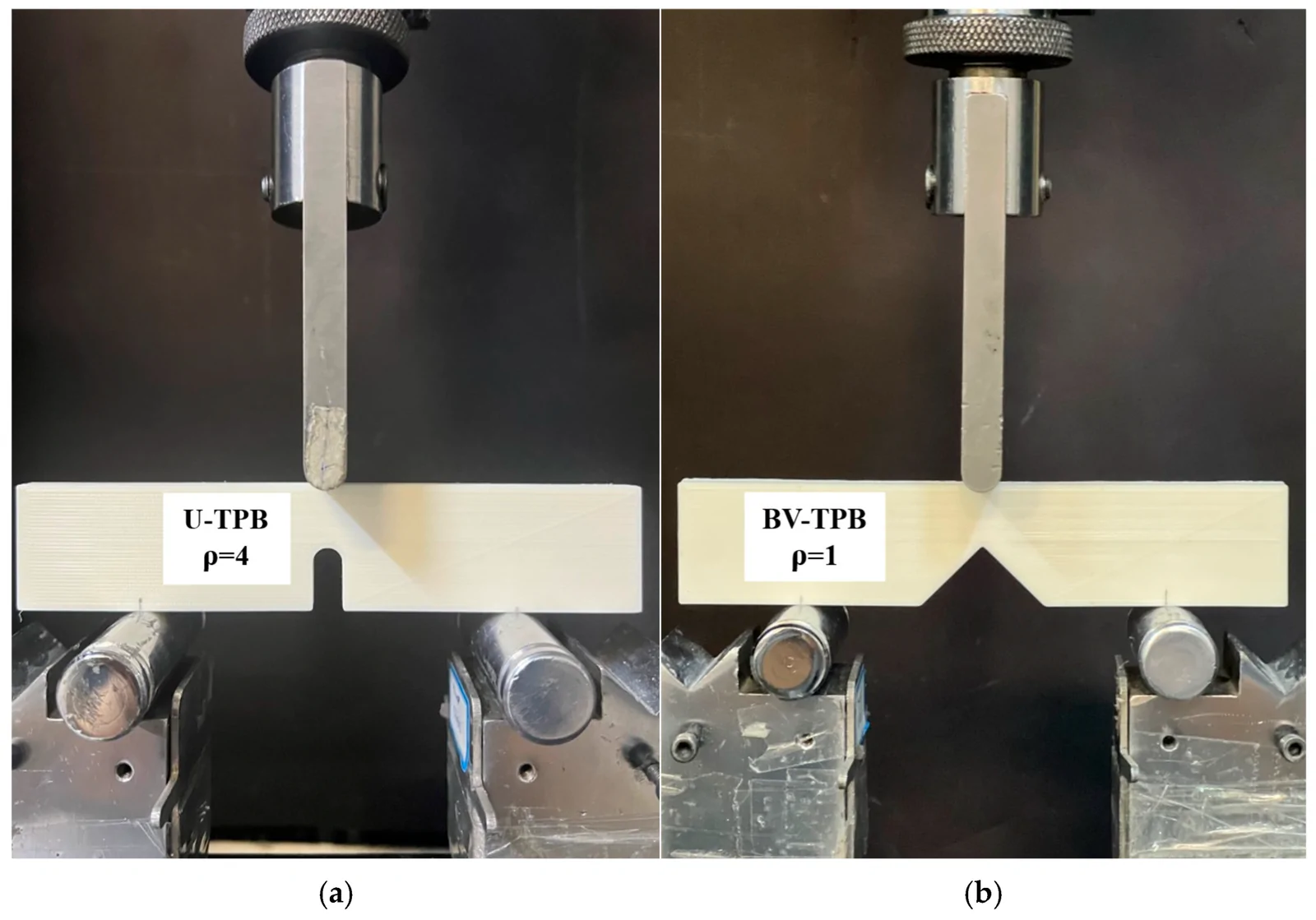
(**a**) U-TPB and (**b**) BV-TPB MEX-printed ABS specimens fabricated with a symmetrical [0/90]s printing architecture.

In terms of test variety, three-point bending has been widely employed for investigating fracture behavior, since it allows relatively direct control and observation of crack initiation and propagation. Accordingly, symmetrical bending tests are carried out on U- and BV-notched TPB specimens to evaluate the fracture loads and characterize their fracture response. Figure 2 displays the three-point bending setup used for the ABS specimens.

Flexural tests are conducted using a SANTAM (STM-150), which is an engineering and design company in Tehran, Iran, universal testing machine with a 5 kN load cell at a fixed crosshead displacement rate of 10 mm/min, as shown in Figure 2. Table 3 presents the flexural test specifications. It should be noted that a single printing architecture (cross-ply) is used for all the printed specimens to ensure that the comparison between the results of the U- and BV-notched specimens is not confounded by variations in the printing architecture.

**Figure 3:**
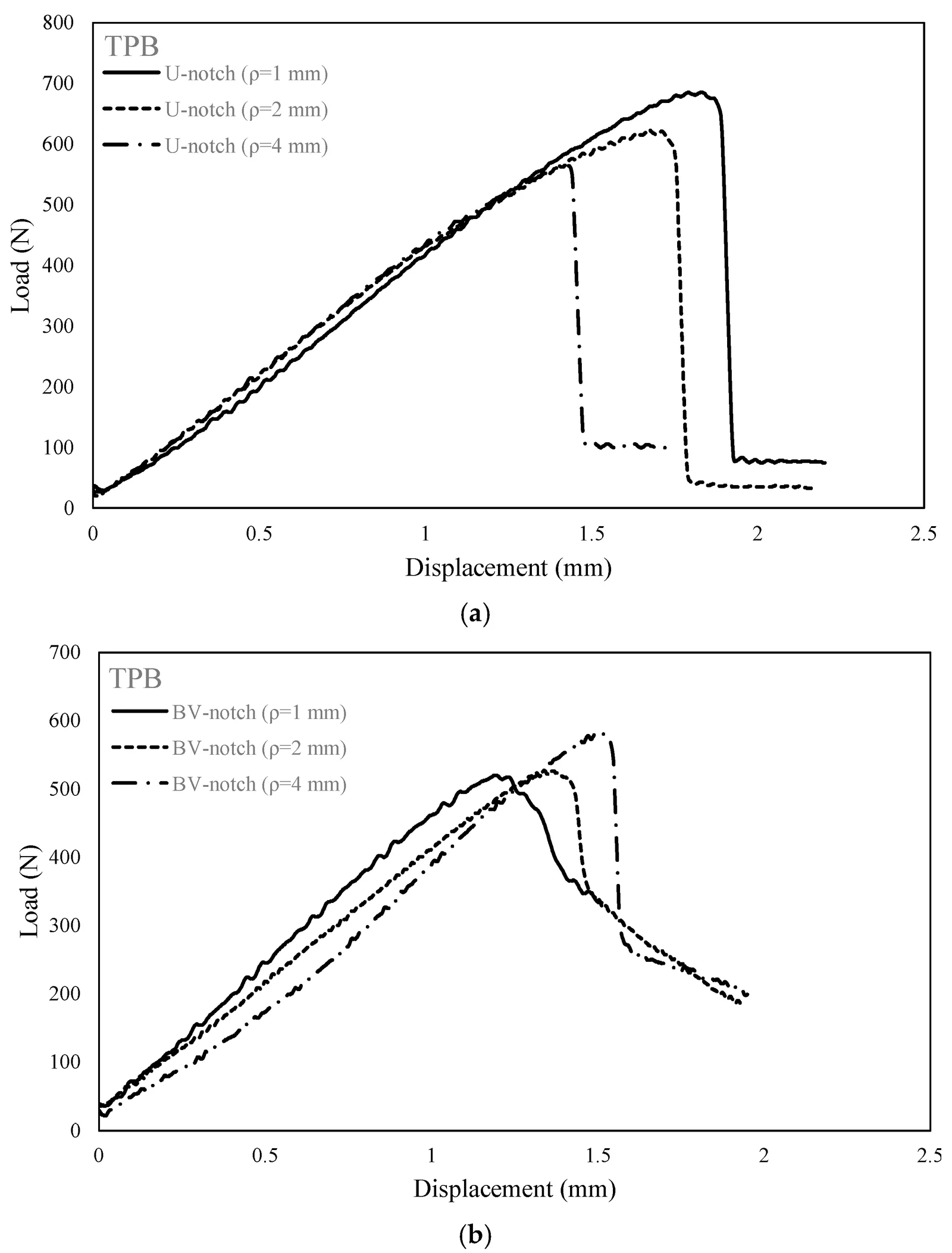
Experimental load-displacement response of the MEX-printed ABS specimens with symmetrical [0/90]_s_ printing architecture, including (**a**) U- and (**b**) BV-notches with different notch root radii.

**Figure 4:**
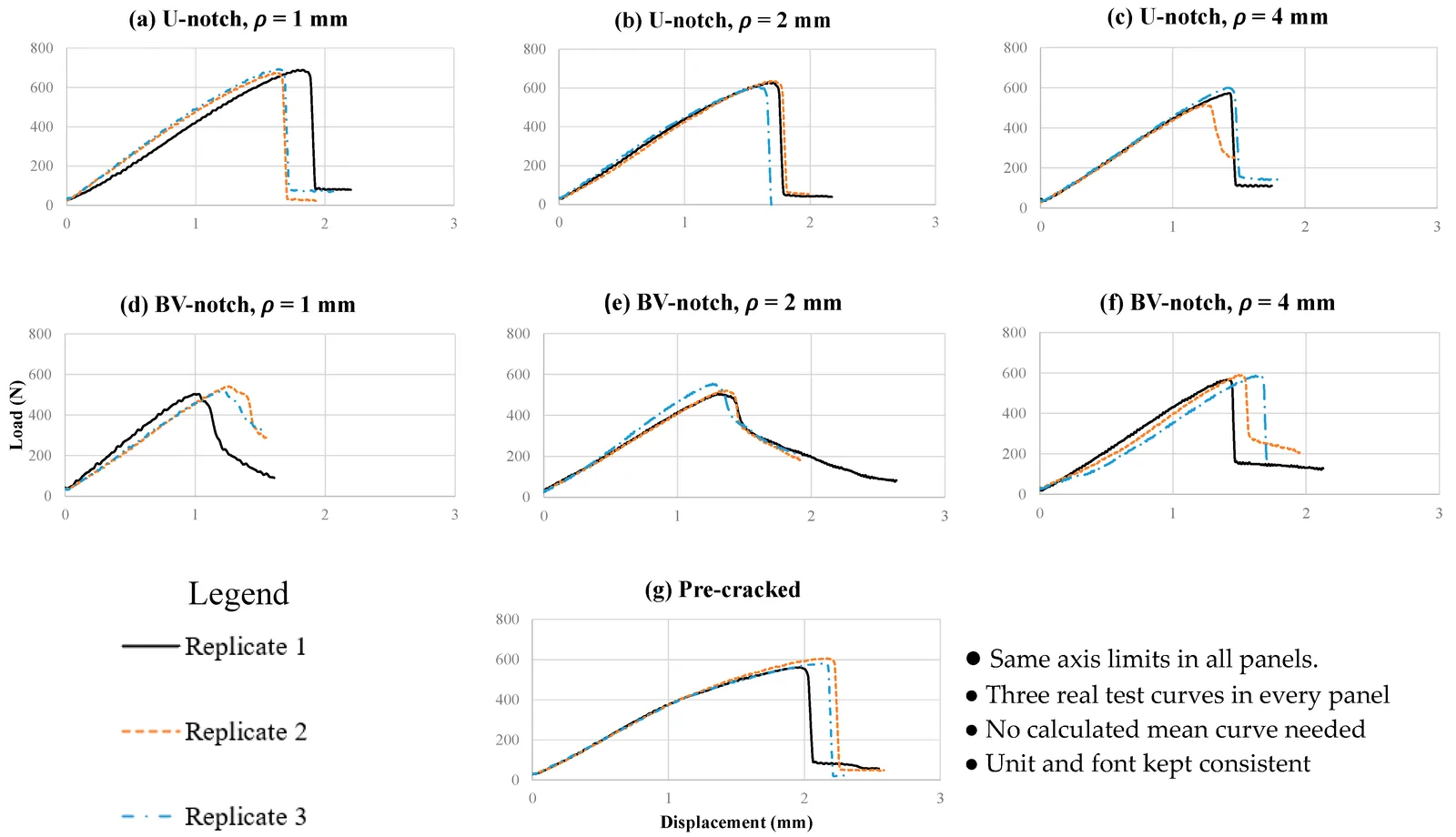
Individual load-displacement curves of the three replicate MEX-printed ABS specimens ([0/90]_s_ printing architecture) tested for each condition: (**a**–**c**) U-notched specimens with ρ = 1, 2, and 4 mm; (**d**–**f**) BV-notched specimens with ρ = 1, 2, and 4 mm; (**g**) pre-cracked specimens.

The bending response of each specimen is continuously recorded to obtain the experimental fracture load. Tests on each notch geometry-radius combination, including the pre-cracked specimens, are repeated three times, giving an average standard deviation of approximately 5%. Figure 3 shows the averaged load-displacement curves for the TPB samples containing U- and BV-notches with varied root radii, and Figure 4 presents the individual curves of all tested specimens, including the pre-cracked ones. The peak load of each curve is taken as the acquired fracture load, and the corresponding values are summarized in Table 4.

A separate finite element model is developed for each specimen configuration to characterize the displacement, stress, and strain energy density (SED) fields in the vicinity of the notch root, which are required for applying the fracture criteria discussed in the following sections.

### 2.4. Finite Element Analysis

The translaminar fracture toughness of the pre-cracked specimen is determined using the commercial finite element (FE) software ABAQUS. Owing to the layer-wise manufacturing process of MEX-printed ABS samples with a cross-ply printing architecture, the cracked sample that contains a pre-existing through-the-thickness crack is expected to undergo translaminar fracture. Since the specimens are printed flat, the crack plane is perpendicular to the deposited layers and intersects all 32 printed layers, corresponding to a translaminar fracture configuration. Due to the alternating 0° and 90° raster directions, the crack may follow across deposited roads or inter-road bonds. Therefore, the fracture resistance is characterized in terms of the translaminar fracture toughness (TLFT), represented by $K_{TL}$, reflecting the combined fracture response of the 0° and 90° layers. The experimental observations also confirm that the type of fracture in the pre-cracked and notched specimens is translaminar rather than interlaminar [41]. The mode I stress intensity factor (SIF), i.e., $K_{I}$, is calculated numerically using the contour integral available in ABAQUS and is subsequently taken as the mode I translaminar fracture toughness ($K_{TL}^{I}$), rather than being calculated from a closed-form $K_{I}$ expression. The computed value of the SIF for the average fracture load of the pre-cracked specimens is 3.16 MPa√m, which is therefore considered equal to TLFT, i.e., $K_{TL}^{I}$= 3.16 MPa√m. It is worth mentioning that the computed SIF corresponds to the average maximum load experimentally obtained for the pre-cracked specimens (583 N), with a variation of approximately 0.04% across ten contours around the crack tip. Considering the experimental variability in the maximum load (SD = 22.6 N), the apparent translaminar fracture toughness is obtained as $K_{TL}^{I}$= 3.16 ± 0.12 MPa√m. Since the exact growth onset of the pre-crack is not independently and precisely monitored during the tests, the calculated SIF value corresponds to the maximum experimental load at/near which the experimental observations confirm the crack growth onset, rather than a confirmed exact crack growth onset load.

**Figure 5:**
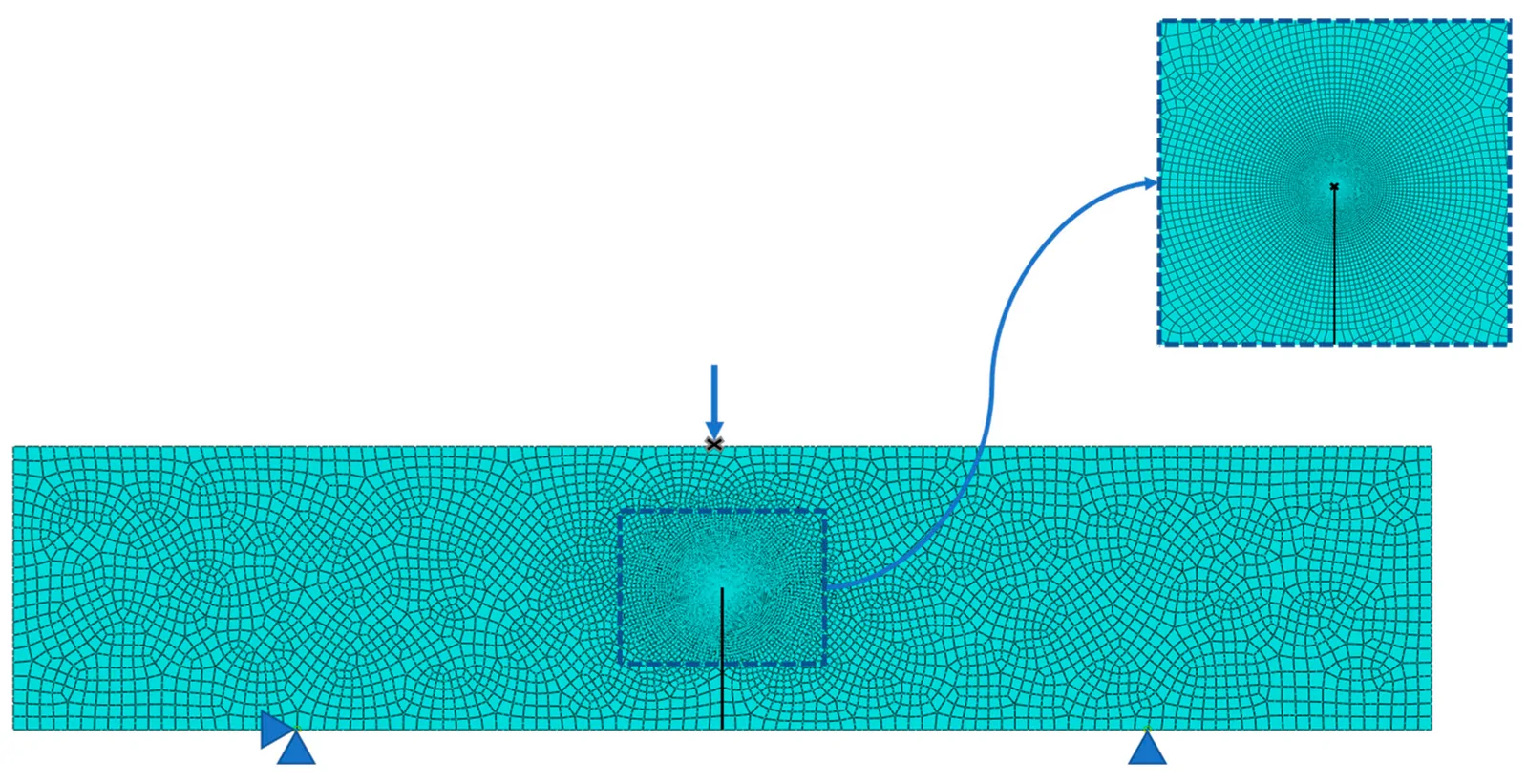
Finite element meshing, loading, and boundary conditions applied to a pre-cracked specimen with a magnified view of the refined mesh pattern near the crack tip. The left support is restricted in both the horizontal and vertical directions, whereas the right one is restricted only in vertical direction.

The pre-cracked samples are modeled as two-dimensional (2D) deformable bodies subjected to three-point bending. The model is discretized using the eight-node biquadratic plane stress elements (Quad 8). Based on the local mesh-independency study reported in [28], an element size of 0.02 mm is selected in the vicinity of the crack tip, providing sufficient numerical accuracy while ensuring convergence of the numerical solution and precise evaluation of the SIF. Figure 5 depicts the finite element discretization of the pre-cracked specimen. The boundary conditions are established by constraining the vertical displacement at both lower supports, while the horizontal displacement is additionally restricted at one support. A concentrated compressive load equal to the average experimentally measured fracture load is also applied to the specimen at the mid-span (see Figure 5).

## 3. Theoretical-Numerical Fracture Prediction Models

The applicability of the VIMC is assessed by comparing theoretical fracture predictions obtained using four different methodologies with the corresponding experimental results. Accordingly, the subsequent subsections describe the governing theoretical framework, finite element procedures, and fracture assessment methodologies adopted in this study.

### 3.1. Virtual Isotropic Material Concept

**Figure 6:**
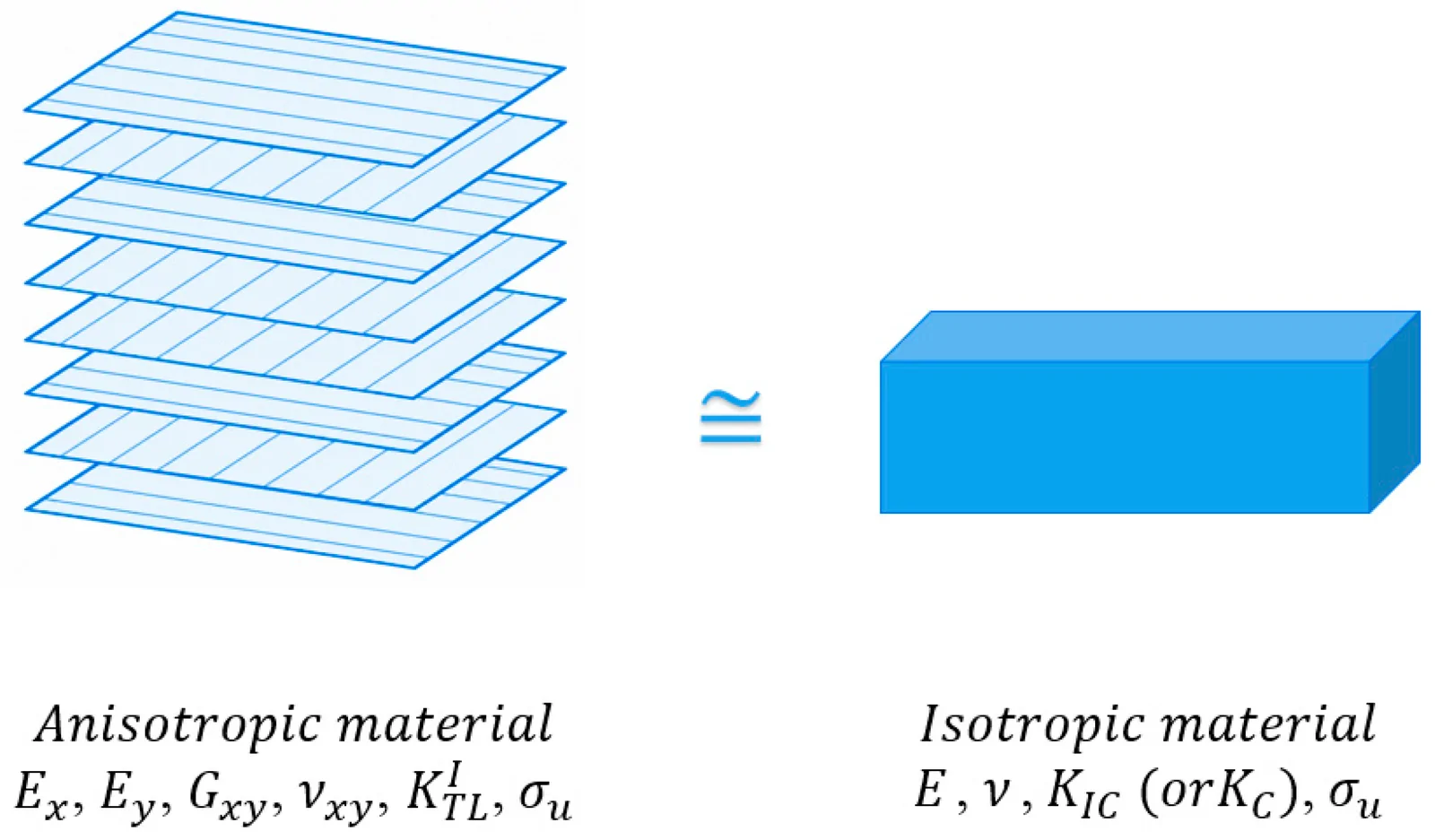
A schematic representation of VIMC.

Within the scope of VIMC, the anisotropic behavior of a MEX-printed material is represented by the equivalent isotropic behavior of a virtual material with the same elastic modulus. This approach eliminates the need for measuring distinct material properties in different directions, such as the longitudinal modulus ($E_{x}$), transverse modulus ($E_{y}$), shear modulus ($G_{xy}$), and in-plane Poisson’s ratio ($\nu_{xy}$). Instead, only two principal fracture-related properties, i.e., the mode I translaminar fracture toughness ($K_{TL}^{I}$) and the ultimate tensile strength ($\sigma_{u}$) must be defined in VIMC-based models. By reducing the anisotropic material representation to an equivalent isotropic description, VIMC provides a simplified framework for fracture and damage assessment without explicitly modeling the individual deposited layers. This approach was introduced by Torabi and Pirhadi [21] as an alternative to conventional layer-by-layer analyses commonly employed for fiber-reinforced composite materials. As previously noted, $K_{TL}^{I}$ and $\sigma_{u}$ are ascertained by testing the cracked TPB and dog bone specimens, respectively. The VIMC could be proficiently linked to the linear elastic notch fracture mechanics (LENFM) criteria to estimate the load-bearing capacity of notched MEX-printed anisotropic specimens. As schematically illustrated in Figure 6, a bulk anisotropic laminated composite that possesses multiple material properties, namely $E_{x}$, $E_{y}$, $G_{xy}$, $\nu_{xy}$, $K_{TL}^{I}$, and $\sigma_{u}$, is assumed to be equivalent to a virtual isotropic plate. The latter is described using the corresponding isotropic properties, i.e., *E* ($E_{x}$ or $E_{y}$ depending on the loading direction), ν, $\sigma_{u}$, and $K_{IC}$. Within the VIMC framework, the fracture toughness of the isotropic surrogate material, i.e., $K_{IC}$, is equated to the $K_{TL}^{I}$. This equivalence provides the capability for fracture assessment and prediction of the load-bearing capacity of MEX-printed components employing established brittle fracture criteria.

In the present study, the symmetrical cross-ply raster architecture consists of equal numbers of layers oriented at 0° and 90°, providing a more balanced distribution of raster orientations than unidirectional printing. Therefore, the in-plane stiffness and strength may exhibit smaller differences between the two principal directions. Moreover, the equivalent elastic modulus and tensile strength used in the VIMC-based models are obtained from specimens with the same raster architecture [39], while the apparent translaminar fracture toughness is determined using the corresponding pre-cracked specimens. Hence, the influence of the printing architecture is indirectly incorporated into the fracture predictions through these material properties. Nevertheless, VIMC remains a simplified representation and does not imply that the printed material is fully isotropic. Furthermore, orthotropic damage modelling is beyond the scope of the present study, as it would require additional characterization of the directional elastic and strength properties.

Due to the layer-wise deposition of material and the presence of inherent voids in MEX-printed components, anisotropic behavior arises. Although anisotropy in such components is less pronounced than in laminated composite materials, its effect remains evident. Hence, VIMC is applied here to the cross-ply MEX-printed ABS material in conjunction with three stress-based and energy-based criteria, along with the extended finite element method (XFEM) coupled with the cohesive zone model (CZM), aiming to estimate the fracture load of rectangular TPB specimens containing U- and BV-notches.

### 3.2. Mean Stress Criterion

**Figure 7:**
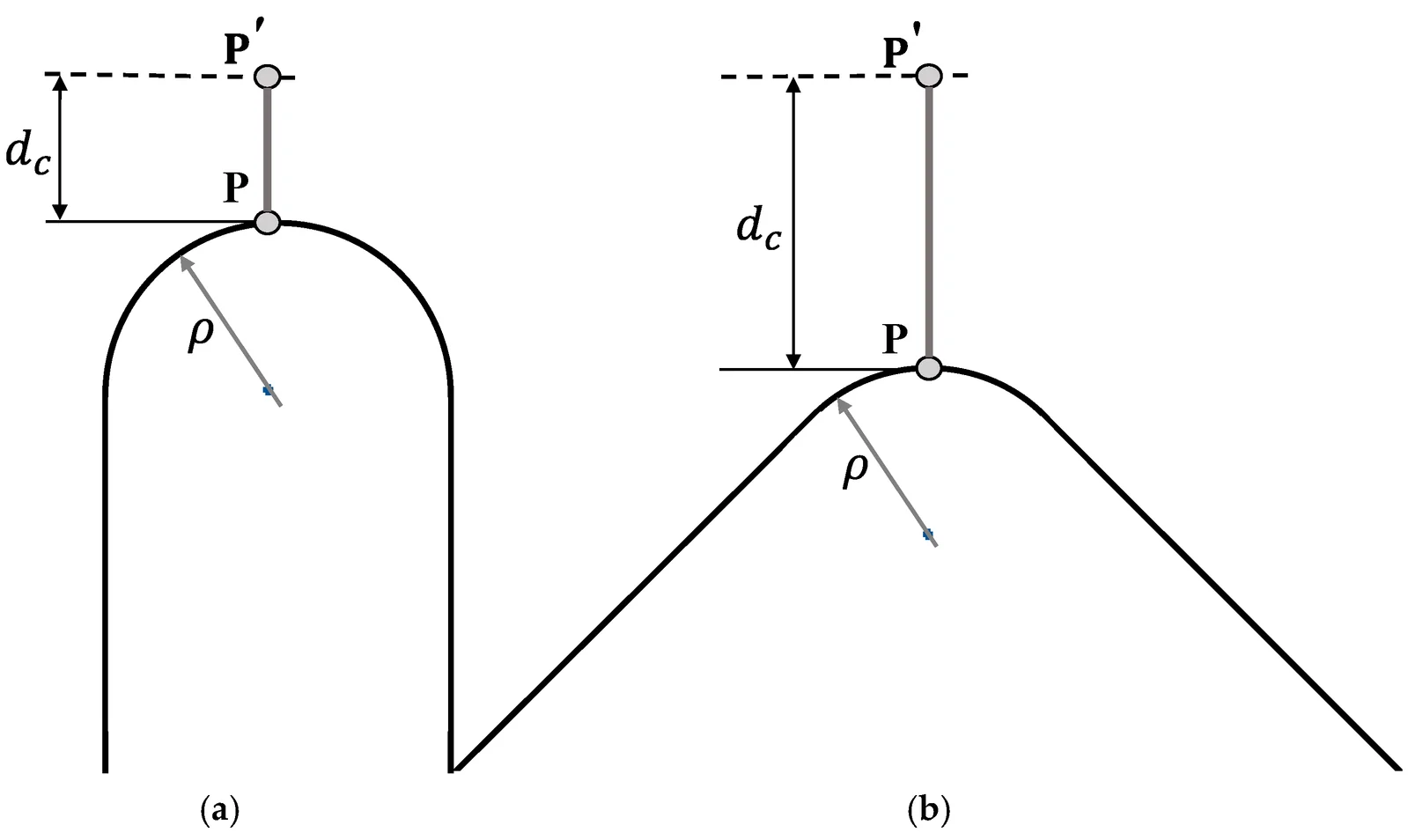
Diagrammatic depiction of the mean stress model for (**a**) U- and (**b**) BV-notches.

In accordance with the MS failure concept, brittle fracture under mode I loading is predicted when the mean circumferential stress ($\bar{\sigma_{\theta \theta }}$), taken precisely over the critical distance $d_{c}$, reaches the material critical stress ($\sigma_{c}$). For materials exhibiting brittle or quasi-brittle behavior, the critical stress is generally considered equal to the ultimate tensile strength ($\sigma_{u}$) [42]. The same consideration is taken in the present study, since the TPB ABS specimens show brittle fracture response. The concepts of the U-MS and BV-MS criteria under pure mode I loading are schematically illustrated in Figure 7.

The critical distance $d_{c}$ is not fitted individually for each specimen, but instead is treated as a material property and obtained from the fracture toughness $K_{IC}$ taken as the $K_{TL}^{I}$, and $\sigma_{c}$ according to:

(1) $$d_{c\;}=\frac{2}{\pi }{\left(\frac{\;K_{IC}}{\sigma_{c}}\right)}^{2}$$

**Figure 8:**
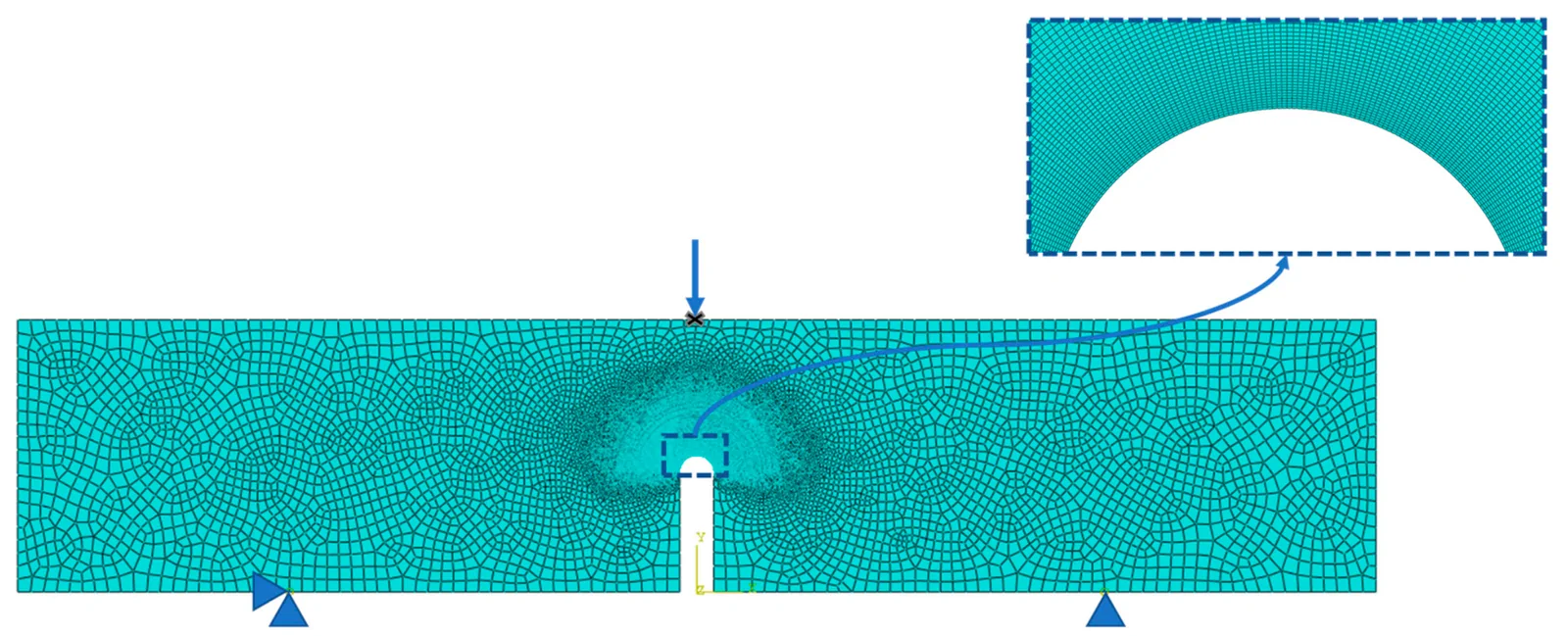
Finite element meshing, loading, and boundary conditions applied to a U-notched specimen with a magnified view of the refined mesh pattern near the notch tip. The left support is restricted in both the horizontal and vertical directions, whereas the right one is restricted only in vertical direction.

To determine the fracture load of TPB ABS notched samples, the FE analyses are carried out in ABAQUS 2022 to obtain the circumferential stress distribution around the notch root. The U-TPB and BV-TPB specimens are modeled under a three-point bending configuration using the boundary and loading conditions already described in the FE modeling section, as shown for the pre-cracked TPB specimens in Figure 5. A prescribed arbitrary load (herein equal to 3000 N) is applied at the loading nose, and the corresponding average circumferential stress over $d_{c\;}$ ($\bar{\sigma_{\theta \theta }}$) is queried directly from the FE results. Furthermore, a mesh size of 0.02 mm is employed in the vicinity of the notch root to achieve sufficient solution accuracy (see Figure 8). The fracture load, $P_{c}$ is subsequently determined by linearly scaling the applied load from:

(2) $$\frac{3000}{P_{c}}=\frac{\bar{\sigma_{\theta \theta }}}{\sigma_{u\;}}$$

### 3.3. Point Stress Criterion

According to the point stress failure model, brittle fracture under mode I loading begins when the circumferential stress ($\sigma_{\theta \theta }$) at the specific critical location ahead of the notch root ($r_{c\;}$) attains the material critical stress ($\sigma_{c}$). As stated before, the material critical stress is assumed equal to $\sigma_{u}$. The critical distance, $r_{c\;}$, is determined from:

(3) $$r_{c\;}=\frac{1}{2\pi }{\left(\frac{\;K_{IC}}{\sigma_{c}}\right)}^{2}$$

**Figure 9:**
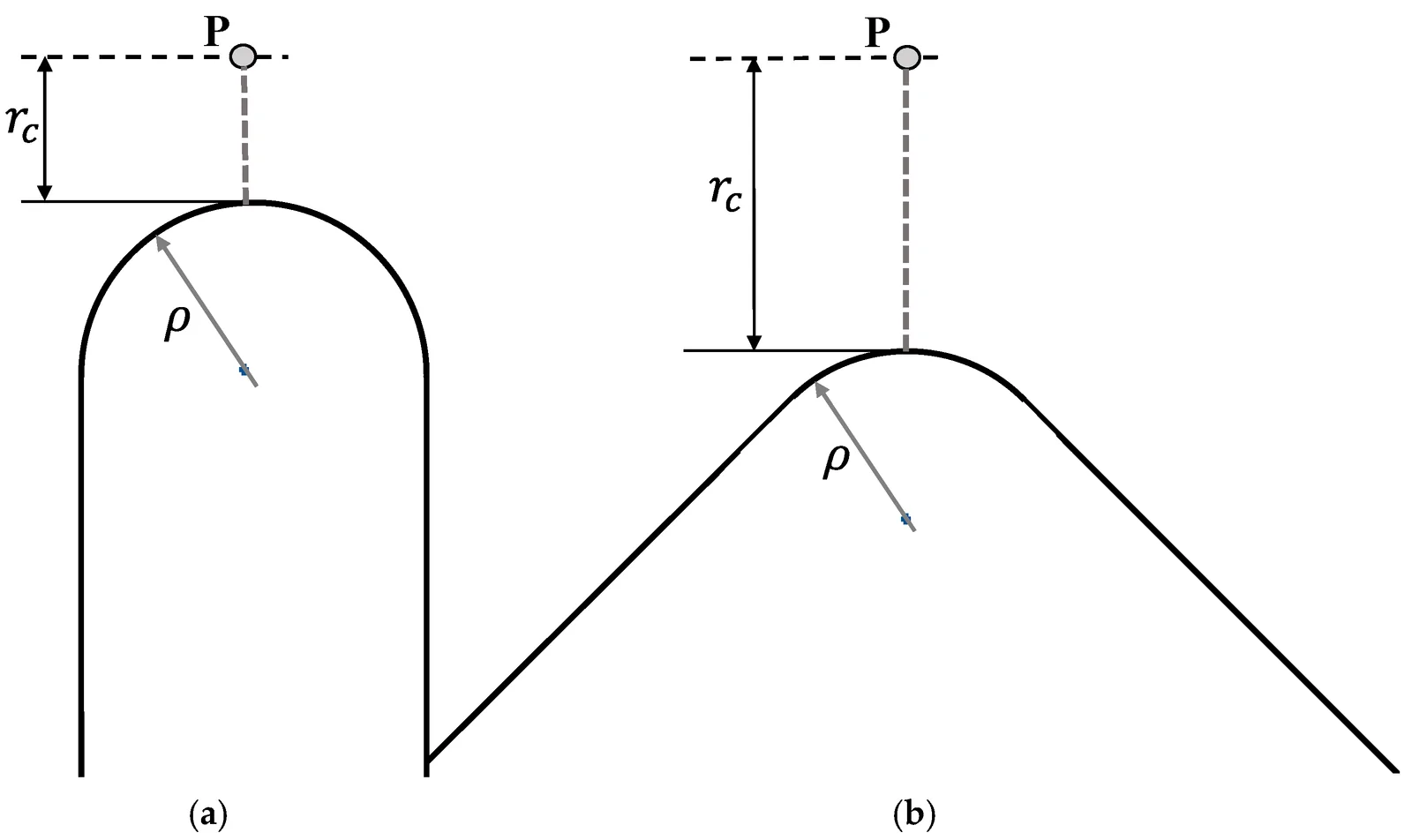
Diagrammatic depiction of the point stress model for (**a**) U- and (**b**) BV-notches.

Figure 9 illustrates the definition of the critical distance of the PS model for both U- and BV-notches under pure mode I loading.

Since the PS and MS approaches operate on the same underlying elastic stress field, the same FE model is employed for this criterion. On this basis, the stress values obtained on the notch bisector corresponding to the prescribed load (3000 N) are simply re-evaluated at $r_{c\;}$ ($\sigma_{\theta \theta }$) rather than averaged over $d_{c\;}$. The fracture load corresponding to the PS approach, i.e., $P_{c}$, is then determined from:

(4) $$\frac{3000}{P_{c}}=\frac{\sigma_{\theta \theta }}{\sigma_{u\;}}$$

### 3.4. Averaged Strain Energy Density Criterion

According to the ASED approach, a crescent-shaped volume of material with the characteristic size of $R_{c}$ surrounding the notch profile is assumed to withstand against brittle failure under mode I loading until the averaged elastic strain energy density (SED) stored within it, denoted by $W_{av}$, reaches the critical SED of the material, i.e., $W_{c}$. This critical energy is regarded as an intrinsic material parameter, and is therefore independent of the notch geometry. The critical SED is obtained from linear elasticity as:

(5) $$W_{c}=\frac{\sigma_{c}^{2}}{2E}$$

**Figure 10:**
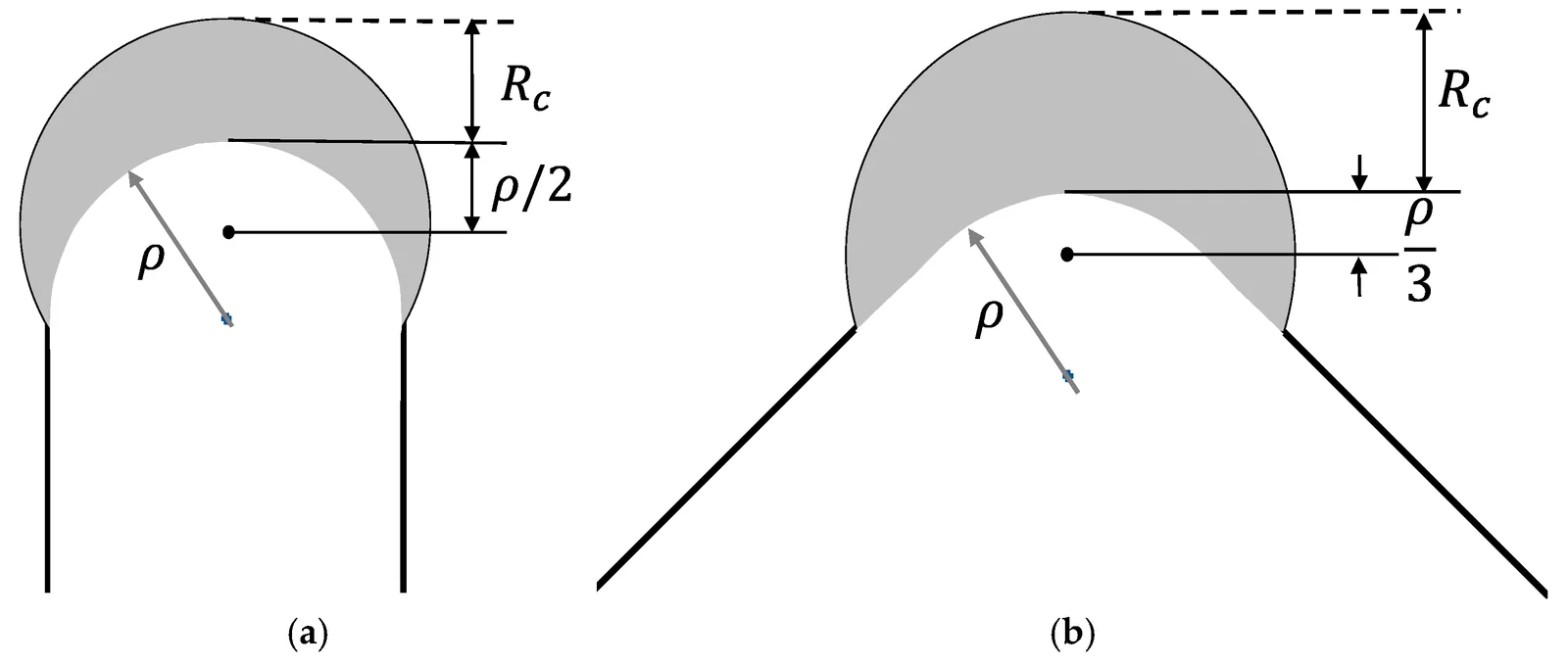
Diagrammatic depiction of the averaged strain energy density model for (**a**) U- and (**b**) BV-notches.

The characteristic size of the volume ($R_{c}$) is related to the fracture toughness and critical stress of the material, and its shape changes with variation in the notch profile. For this reason, the volume does not sit at the same center for both notch types. For U-notches, it is centered at a distance ρ/2 behind the notch root, while for BV-notches with a 90° opening angle, the distance becomes equal to ρ/3, reflecting the sharper local curvature. Figure 10 demonstrates the distinction between both geometries.

Since the TPB specimens can be regarded as thin, comparing the thickness with the in-plane dimensions, the plane-stress expression of $R_{c}$ can be used here as follows [43]:

(6) $$R_{c}=\frac{5-3\nu }{4\pi }{\left(\frac{K_{IC}}{\sigma_{c}}\right)}^{2}$$

Application of the ASED criterion to the TPB specimens follows the same general FE procedure used for the two stress-based methods above, except the crescent-shaped volume should first be partitioned within the FE model for each notch geometry, after which the corresponding averaged SED ($W_{av}$) is extracted under a prescribed load (3000 N). The fracture load, $P_{c}$, can subsequently be estimated as:

(7) $$\frac{3000}{P_{c}}=\sqrt{\frac{W_{av}}{W_{c}}}$$

The critical parameters corresponding to all three criteria, including $r_{c\;}$ and $d_{c\;}$ for stress-based models, and $R_{c}$ together with $W_{c}$ for the ASED model, are summarized for the MEX-printed ABS notched specimens in Table 5.

### 3.5. Extended Finite Element Method Incorporating the Cohesive Zone Model

Predicting the crack initiation location and subsequent propagation path remains a major challenge in fracture analysis of engineering structures, particularly when the crack trajectory is not known a priori. The extended finite element method (XFEM) facilitates predicting the fracture load and path by enriching the degrees of freedom of elements that lie in the potential cracking domain. This approach enables the discontinuity to be directed within the elements by obviating the need to prescribe the crack path or to use extremely fine meshes around every notch or stress concentrator, and subsequently re-meshing the crack-tip zone to update it as the crack grows. Once XFEM is implemented, the actual onset and growth of the crack can be governed by an appropriate damage model. In the present study, XFEM is paired with the linear elastic cohesive zone model (CZM) to predict the fracture load of TPB ABS specimens.

Based on the physical idea behind the CZM, the material ahead of the notch root or crack does not fail instantaneously; instead, material degradation occurs through a fracture process zone (FPZ) until the true physical crack forms. According to CZM, when the cohesive traction (*T*) exceeds the critical stress ($\sigma_{c}$), typically being identical to the material’s ultimate tensile strength ($\sigma_{u}$), the initiation of failure in the FPZ occurs. The cohesive traction and virtual crack separation displacement (δ) are correlated as:

(8) $$T=f\left(\delta \right)$$

**Figure 11:**
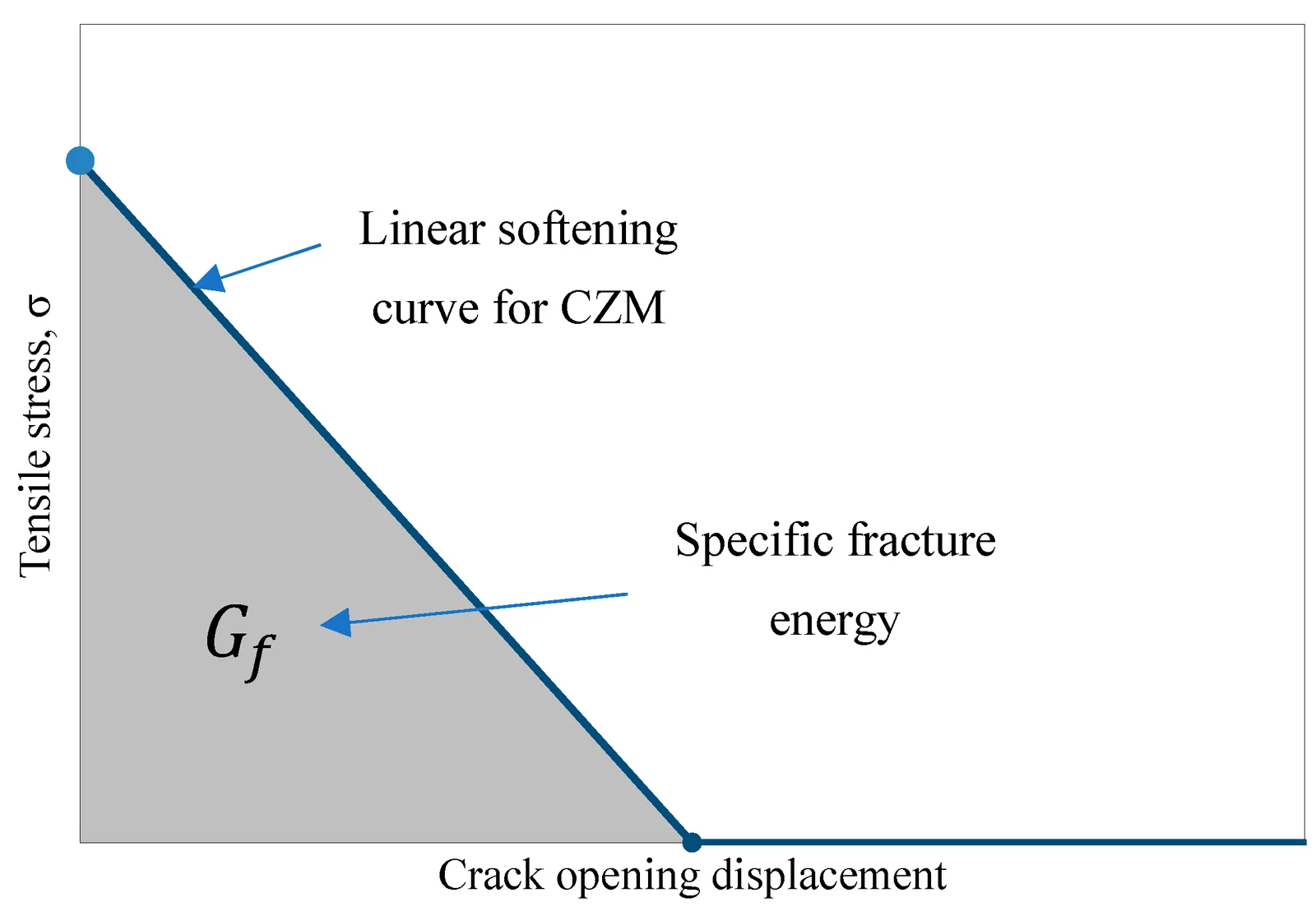
Illustration of the linear tension-softening law in the cohesive zone model.

The softening function, *f*, is specified as a material input in the FE solver that describes how the cohesive traction progressively degrades as the virtual crack separation displacement (δ) increases. Since the cross-ply ABS material behaves in a brittle, linear elastic manner, a linear softening function is simply adopted in the present analysis. As shown in Figure 11, the area under the softening curve corresponds to the fracture energy, $G_{f}$, which controls the rate of damage evolution and, together with the ultimate tensile strength, $\sigma_{u}$, is supplied as a material property input to the FE model. The fracture energy can be calculated using the following equation:

(9) $$G_{f}=\frac{K_{IC}^{2}}{E}$$

Because the TPB specimens are relatively thin, the plane-stress formulation is employed, yielding a fracture energy of 8.58 N/mm for the cross-ply ABS material.

In addition, damage initiation is governed by the maximum principal stress (Maxps) criterion implemented in ABAQUS. According to this criterion, cracking at the notch initiates when the maximum principal stress ($\sigma_{p}$) attains $\sigma_{u}$, after which the crack propagates. This condition is expressed as follows:

(10) $$\frac{\sigma_{p}}{\sigma_{u}}=1$$

To maintain manageable computational cost, a two-dimensional plane-stress model is adopted. Because the notch root sits at the location of maximum principal stress under bending, crack initiation is expected to begin at the notch root. The crack then propagates upward from the notch root through the material toward the loading nose until full separation. The region ahead of the notch, spanning from the notch root to the top surface, where the crack growth is expected, is known as the XFEM fracture zone. This region is meshed using the four-node bilinear plane-stress quadrilateral elements, with the element size progressively shrinking as the mesh approaches the notch root, thereby refining the resolution where the stress gradient is very high.

**Figure 12:**
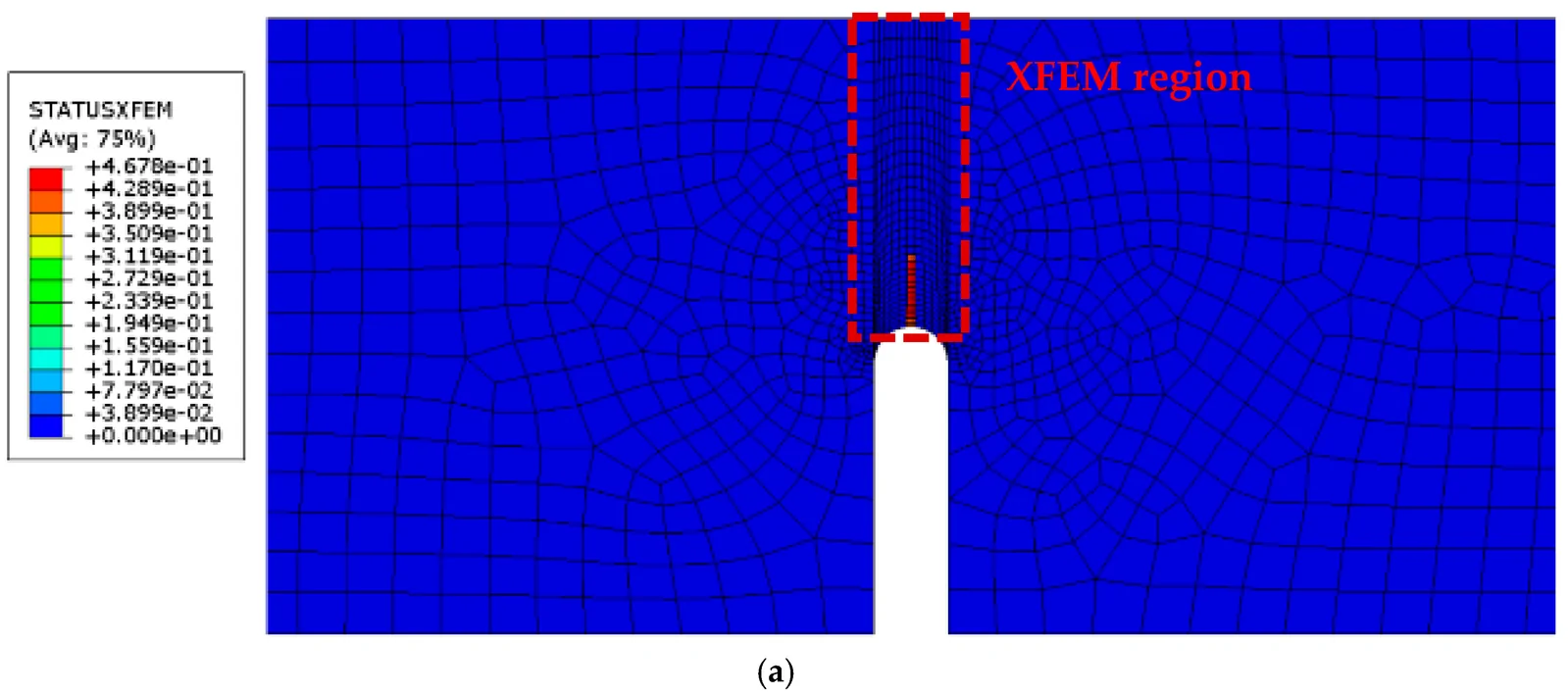

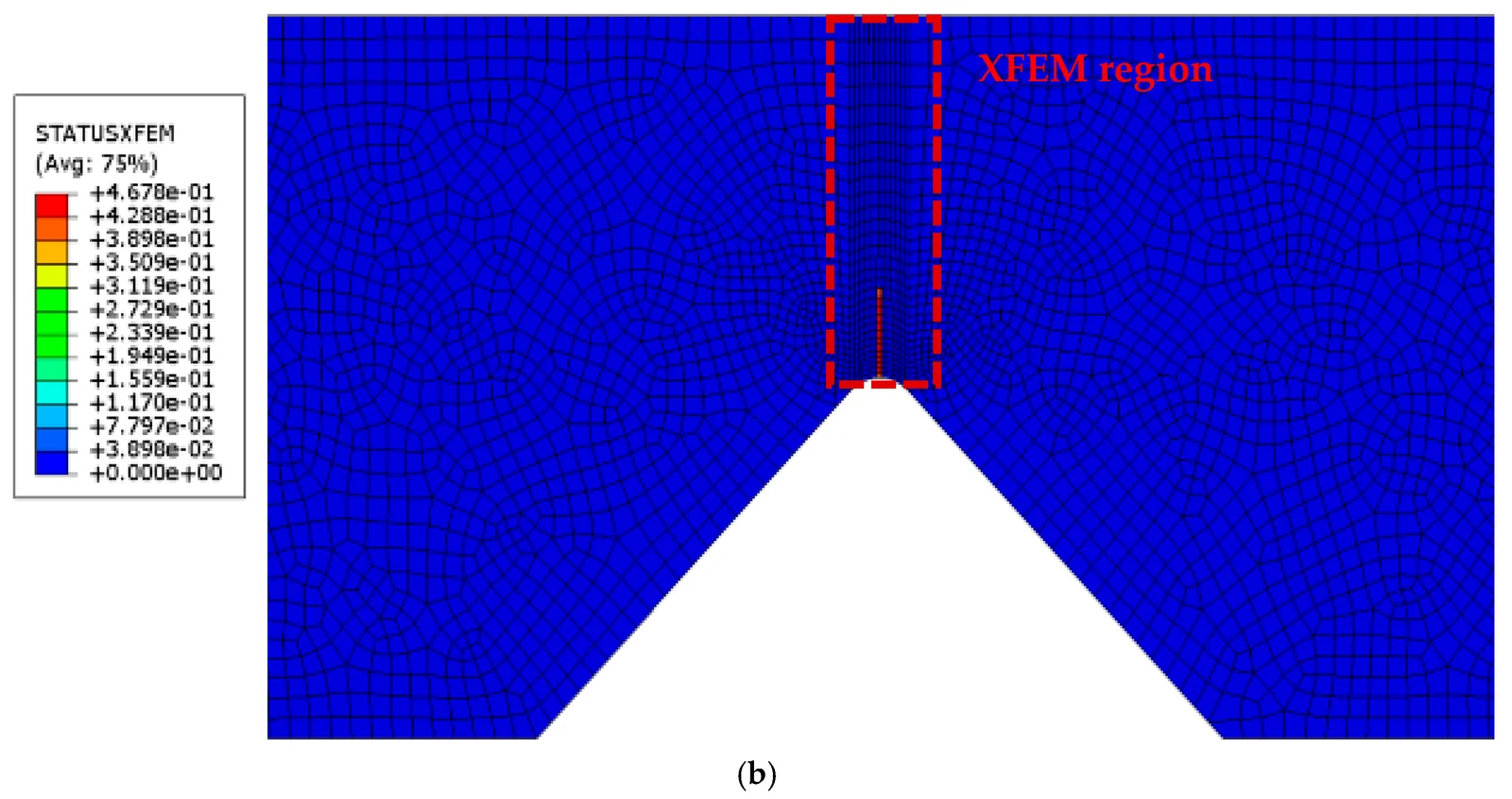
Zone of analysis for the extended finite element method along with the associated grid configurations for (**a**) U- and (**b**) BV-notches; the red lines represent the crack emanating from the notch root.

The boundary conditions are consistent with those described in the preceding sections, except that an imposed displacement is applied to the mid-span on the compression face instead of a concentrated force. The resulting numerical load-displacement curve is obtained, and the peak of this curve is taken as the load-bearing capacity of the specimen. Notably, in the FE model, the STATUSXFEM field output, which is an element crack-status variable, is used for crack-status visualization; see Figure 12, where a value of unity means the element is completely cracked. It should be noted that the validation of XFEM-CZM-VIMC is conducted herein only by means of comparing the predicted fracture load with the experimentally achieved one, not by comparing the predicted crack growth path with the corresponding experimentally observed path, because no crack path measurements are made. Therefore, the accuracy of the crack trajectories obtained from the XFEM-CZM-VIMC simulations remains experimentally unverified, and direct experimental tracking of crack evolution is needed in future works.

To evaluate the influence of mesh size on the XFEM-CZM-VIMC predictions, a mesh-sensitivity analysis is performed by varying the local element size around the notch root. The results show relatively small differences in the predicted fracture loads among the mesh configurations that achieve numerical convergence and produce the expected crack propagation behavior. However, the mesh size affects the numerical stability of crack initiation and propagation. Excessively fine meshes cause convergence difficulties in some simulations, whereas coarse meshes provide insufficient resolution of the stress gradients near the notch root. Among the successfully converged mesh configurations, the predicted fracture loads vary by approximately 3%. Accordingly, meshes containing 13 to 19 elements along the notch boundary are selected, depending on the notch geometry and root radius. These meshes provide stable numerical solutions, with crack initiation at the notch root and subsequent propagation along the specimen symmetry plane.

## 4. Results and Discussion

The structural response of notched TPB ABS specimens subjected to three-point bending provides insight into the fracture mechanism of MEX-printed material in the presence of stress concentration and opening-mode loading. For this reason, the experimental fracture loads are discussed together with the predictions obtained from the stress-based, energy-based, and numerical-damage-based approaches, considering the layered nature of 3D-printed parts, in which the loading condition, geometry, and material anisotropy may have significant influences on the fracture behavior. Experimental load-displacement records confirm an almost linear-elastic behavior culminating in predominantly brittle fracture. This behavior is consistent with that reported for the same [0/90]_s_ cross-ply ABS material under tensile loading [39].

The experimental fracture loads summarized in Table 4 reveal a contrasting fracture response depending on the notch profile. For the U-notched configurations, the fracture load decreases as the notch root radius increases, whereas the BV-notched specimens do the reverse, gaining load-bearing capacity as the radius increases. The latter behavior agrees with the expected general trend that a blunter notch lowers the stress concentration and consequently raises the load required for fracture [44]. The U-notched specimens, however, run counter to this conventional expectation, which is attributed to the stress distribution in these specimens and the relatively great critical distance of the MEX-printed ABS material.

A Two-way analysis of variance (ANOVA) is performed on the fracture loads reported in Table 4, which considers the notch profile and notch root radius as independent factors. Tukey pairwise comparisons are performed using the combined statistical analysis of the ANOVA. The notch profile and its interaction with notch root radius are significant (*p* < 0.001), whereas the main effect of notch root radius is not (*p* = 0.079). The radius effect within each profile is significant for both the U-notched (*p* < 0.001) and BV-notched (*p* = 0.012) specimens. In the pairwise comparisons, the decrease in the U-notch load between 1 and 4 mm is significant (*p* < 0.001), whereas the differences between adjacent radii are not. For BV-notch, the fracture load increases between ρ = 2 and 4 mm, although none of the pairwise comparisons reach statistical significance (*p* > 0.05). Furthermore, the fracture loads of U- and BV-notched specimens differ significantly at ρ = 1 and 2 mm, but not at ρ = 4 mm. These findings confirm a significant interaction between the notch profile and root radius, with stronger pairwise evidence for the decreasing U-notch fracture load than for the increasing BV-notch fracture load.

**Figure 13:**
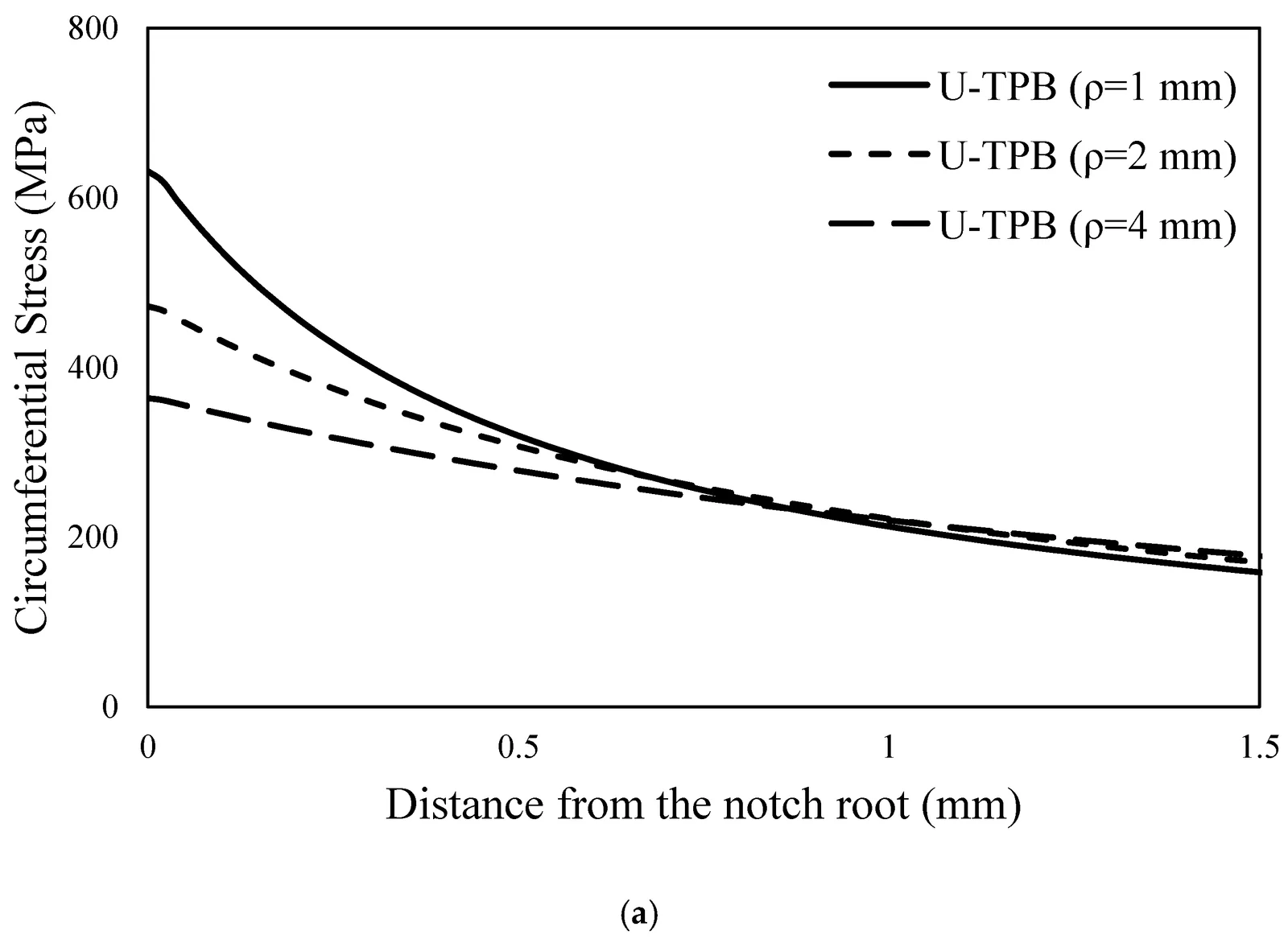

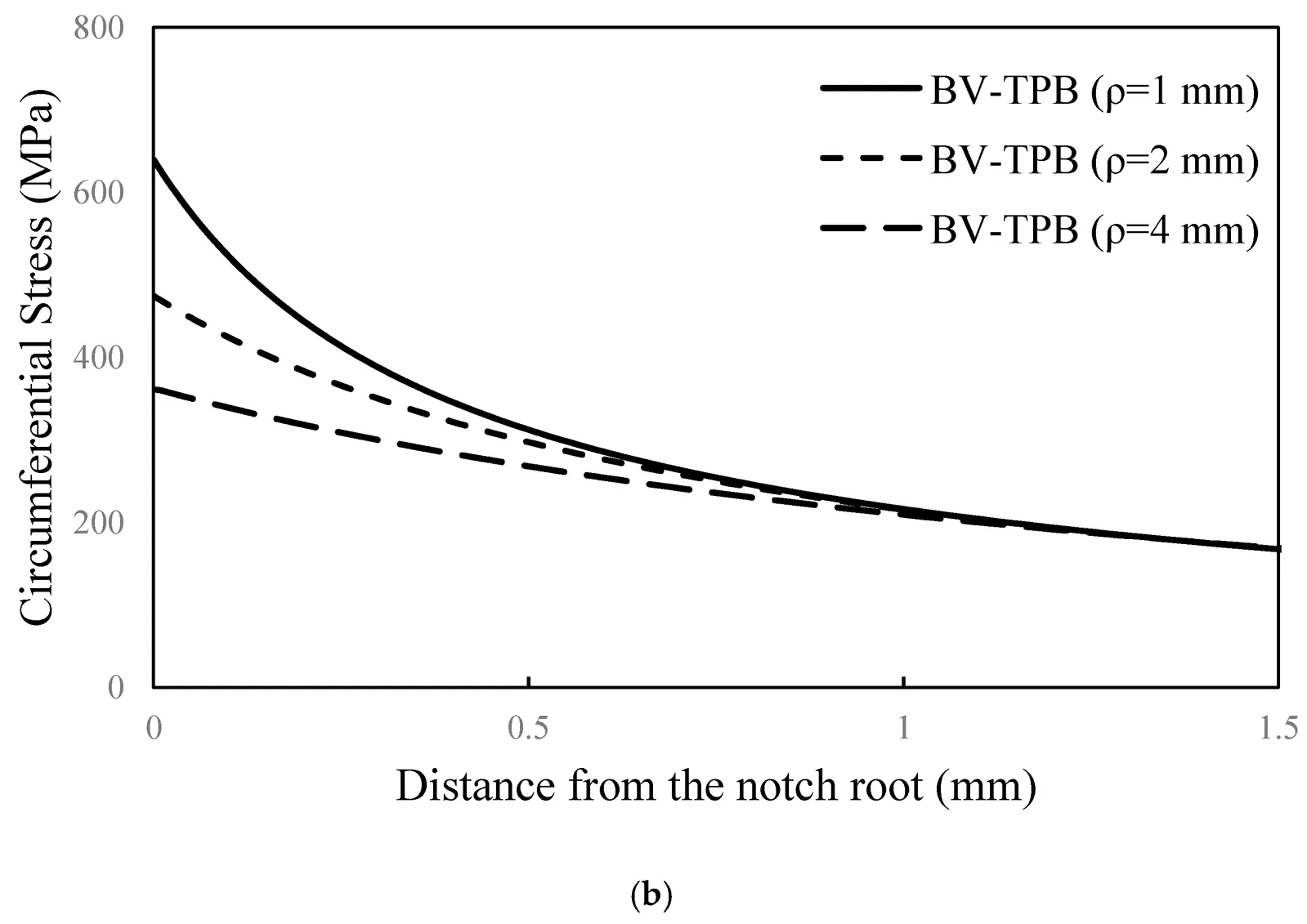
Circumferential stress variation with distance ahead of the notch root for TPB ABS specimens with (**a**) U-shaped and (**b**) BV-shaped notches under the load of 3000 N.

The trend of the fracture load variation in U-notched specimens can be interpreted using the stress distributions illustrated in Figure 13a, where decreasing the notch root radius sharply increases the circumferential stress near the notch root, but the rate at which this stress decays with distance differs among the three radii. The specimen with a 1 mm root radius carries the highest stress directly at the notch root, yet this stress falls off more rapidly with increasing distance from the root. On the other hand, a larger radius produces a lower peak stress at the root itself, but the circumferential stress field extends over a greater distance before decaying. At the material critical distance (*r_c_* = 1.2 mm), while the stress values for the three root radii are very close together, the stress increases as the root radius increases from 1 mm to 4 mm. This simply means that almost the same fracture loads are expected for the three root radii, with a slight decrease in the fracture load as the notch root radius increases. This observation indicates that fracture assessment based solely on the stress at the notch root cannot represent the actual fracture conditions, but instead incorporating the theory of critical distances (TCD) is necessary. A similar trend can also be seen in Figure 13b for BV-notched specimens, except that the stress at the critical distance slightly decreases as the notch root radius increases, leading to a slight increase in the fracture load with increasing root radius. It is worth mentioning that the notch root radius alone does not completely characterize the fracture phenomenon in notched members. The same value of root radius for the U- and BV-notched specimens generates different spatial stress distributions because of their different opening angles, particularly under three-point bending, where the nominal stress itself varies through the specimen width. Consequently, the fracture response appears to be governed by the combined effects of notch root radius, notch profile, stress gradient, and the characteristic length of the material. These numerical stress distributions suggest a possible explanation for the experimentally observed decrease in fracture load with increasing U-notch root radius, rather than being a proven physical explanation.

The microstructure of MEX-printed ABS may also contribute to the specific failure process. The specimens are printed flat, so the crack plane is perpendicular to the deposited layers. Layers comprise 0° and 90° raster directions; thereby, half of them have roads perpendicular to the crack plane, which the crack must cross, whereas in the other half the roads are oriented parallel to it and the crack can follow the weaker inter-road bonds. The importance of interfacial characteristics in fracture behavior has also been reported in dissimilar superalloy joints produced by inertia friction welding, where variations in the weld microstructure influenced the fracture location and crack propagation behavior [45]. In addition, even at 100% infill density, the inherent voids and imperfect bonding remain, as observed in the same material under tension [39], and they are a source of unavoidable scatter [4,16,39]. The critical distances of the material correspond to several road widths (about 2.5 to 10 roads based on the extrusion width of 0.48 mm), so the fracture process zone (FPZ) contains many roads and microstructural defects. A larger notch root radius enlarges the highly stressed region, which may then contain more voids and weak bonds; this may contribute to the lower fracture load of the U-notched specimens at ρ = 4 mm, consistent with the largest scatter observed for this case. However, this microstructural argument cannot explain the opposite tendency of the BV-notch. A possible reason is that, for an equal radius, the control volume around the BV-notch is smaller than that around the U-notch (Table 5), so it contains fewer microstructural defects, and other factors may play the main role in its fracture behavior.

**Figure 14:**
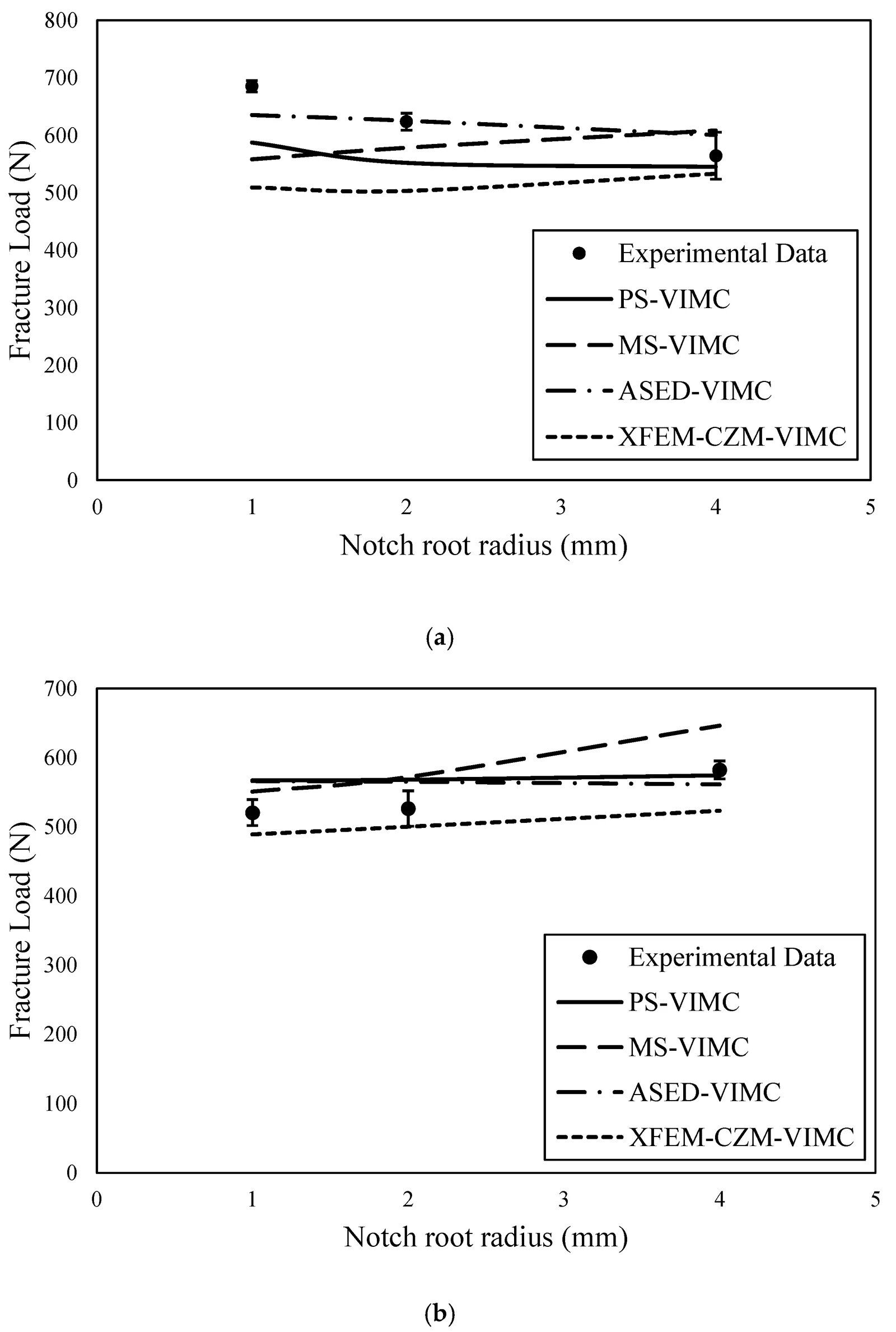
Effect of notch root radius on the experimentally measured and theoretically-numerically predicted fracture loads of TPB cross-ply ABS specimens for (**a**) U-notched and (**b**) BV-notched specimens.

Figure 14 illustrates the dependence of the fracture load on the notch root radius for both the experimental measurements and theoretical-numerical predictions. The theoretical-numerical fracture loads obtained from the four VIMC-based criteria are listed in Table 6 together with the experimental mean discrepancies, and their percentage discrepancies from the experimental means are given in Table 7, allowing assessment of how well VIMC performs once linked to each criterion under a bending-dominated stress state. The mean absolute percentage discrepancy (MAPD) is then defined as:

(11) $$d_{i}=\frac{\left|P_{exp}-P_{theory}\right|}{P_{exp}}$$

where $P_{\exp}$ and $P_{\mathrm{theory}}$ denote the experimentally obtained and theoretically predicted fracture load values, respectively.

Overall, for the symmetrical cross-ply ABS notched specimens investigated within the 1–4 mm notch root radius range under symmetrical three-point bending, all four approaches provide reasonable estimates of the mean experimental fracture loads, with average differences ranging from 5.7% to 11.5%. The ASED-VIMC criterion exhibits the lowest average percentage difference of 5.7%, followed by the PS-VIMC and MS-VIMC criteria with average differences of 7.4% and 10.1%, respectively, while the XFEM-CZM-VIMC criterion trails behind at 11.5%, presenting the largest deviation as the most conservative criterion. Although the overall differences between the three stress- and energy-based criteria are relatively small, their predictive performance varies considerably with the notch profile.

For the specific conditions investigated, the ASED-VIMC criterion is almost unaffected by the notch geometry, with an average difference of 5.7% for both U-notched and BV-notched specimens. PS-VIMC criterion shows a moderate sensitivity to notch type, whereas the MS-VIMC and XFEM-CZM-VIMC criteria show greater sensitivity. This behavior is also observed in Figure 14, where the nearly zero slopes in the ASED-VIMC criterion indicate that the notch root radius has a limited effect on predicted fracture load.

XFEM-CZM-VIMC criterion represents a different framework because this criterion does not rely on a specific critical distance or control volume. Its difference pattern reflects the sensitivity to the damage-softening law and mesh discretization adopted in the cohesive zone model, rather than stress distribution surrounding the notch border. The relatively high differences obtained for some U-notched configurations, particularly with the 1 and 2 mm root radii, suggest that the numerical damage formulation is more sensitive to variations in notch geometry compared with the other criteria. Nevertheless, it still provides reasonable predictions with an overall average difference of 11.5%. As seen in Figure 13, the XFEM-CZM-VIMC criterion is the most conservative criterion among the four criteria. Perhaps, the main reason for this is that the cohesive zone model in ABAQUS software takes into account the maximum principal stress value at the notch root as one of the governing parameters to realize the notch cracking. Since it does not involve the critical distance, at which the stress considerably decreases with respect to the notch root, it predicts a lower load for fracture to take place. Most likely, adding a user-material (UMAT) to ABAQUS that considers the principal stress at the critical distance as a constraint for crack initiation from the notch root may result in a more accurate estimation of the fracture load by the XFEM-CZM-VIMC criterion.

As reported in previous studies [23,28,39], the MS-VIMC criterion has generally provided the most accurate fracture predictions. The present results do not fully reproduce this general trend, since the MS-VIMC criterion exhibits a higher average deviation compared with the PS-VIMC and ASED-VIMC criteria for the investigated TPB specimens. This difference can be partially attributed to the distinct stress distribution generated by the present notch and specimen geometries under bending-dominated loading, meaning that the accuracy is influenced by how appropriately the selected critical distance represents the actual fracture process zone.

To evaluate the influence of the assumed stress state on the predicted fracture loads, the plane-stress formulation adopted in this study is further examined through the dimensional requirements of ASTM D5045 [46] and additional plane-strain numerical analyses. According to ASTM D5045, the specimen thickness (B), crack length (a), and remaining ligament (W − a) must satisfy the following criterion for a valid plane-strain fracture-toughness measurement:

(12) $$B,\;a,\;(w-a)\geq 2.5{\left(\frac{K_{TL}^{I}}{\sigma_{y}}\right)}^{2}$$

where $\sigma_{y}$ is the tensile yield stress or, for materials exhibiting brittle fracture, the tensile stress at fracture. Using the apparent fracture toughness of 3.16 MPa√m and the maximum tensile stress of 36.6 MPa reported in [39], the calculated dimensional requirement is approximately 18.6 mm. However, the present specimens have a thickness of 8 mm, while the crack length and remaining ligament are both 16 mm. Therefore, the dimensional requirements for a valid plane-strain fracture toughness measurement are not satisfied. Nevertheless, this dimensional assessment alone does not establish a plane-stress condition in the notched specimens.

To further examine the influence of the stress state assumption, additional plane-strain analyses are performed for representative U- and BV-notched specimens with a notch root radius of 4 mm. For the ASED-VIMC criterion, the plane-strain strain energy density formulation is adopted, resulting in a critical radius of 1.81 mm, compared with 2.36 mm under plane stress. As summarized in Table 8, the predicted fracture loads decrease by 5.2% and 3.9% for the U- and BV-notched specimens, respectively.

For the XFEM-CZM-VIMC criterion, the predicted fracture loads show smaller differences between the two formulations, with variations of 0.2% for the U-notch and 0% for the BV-notch. However, the fracture energy is also adjusted from 8.58 to 7.59 N/mm for the plane-strain analyses. Therefore, these differences reflect the combined influence of the stress state formulation and the corresponding fracture energy parameter.

Overall, the additional analyses indicate that the two formulations produce relatively small differences in the predicted fracture loads for the selected configurations, although the differences are more noticeable for the ASED-VIMC criterion.

## 5. Conclusions

This study investigated the accuracy of theoretical-numerical predictions of the notch strength of printed ABS by taking the MEX-induced anisotropy into consideration using VIMC integrated with four distinct failure models, i.e., the PS, MS, and ASED criteria, along with the XFEM-CZM model, under a bending scenario. The single-edge U- and BV-notched TPB specimens, fabricated from ABS with a symmetrical cross-ply configuration, were tested at a cross-head displacement rate of 10 mm/min. The experimental results demonstrated a distinct dependence of fracture load on notch profile. Increasing the notch root radius from 1 to 4 mm reduced the fracture load of the U-notched specimens, whereas the BV-notched specimens exhibited an increasing trend in fracture load with increasing radius. This contrasting response was attributed to the interaction between the notch geometry, bending stress field, and material characteristic length. All four VIMC-based approaches provided reasonable predictions of the mean experimental fracture loads with overall average differences between 5.7% and 11.5%. In descending order of predictive accuracy, the methods were ranked as follows: ASED-VIMC (5.7%), PS-VIMC (7.4%), MS-VIMC (10.1%), and XFEM-CZM (11.5%). Among the investigated criteria, the ASED-VIMC criterion provided the closest overall agreement with the experimental fracture loads within the investigated notch root radius range (ρ = 1–4 mm) and symmetrical [0/90]_s_ printing architecture under symmetrical three-point bending, exhibiting almost identical accuracy across both notch types examined. This behavior highlighted the advantage of an area-averaged parameter for the two notch types investigated. However, further investigations involving different notch opening angles, printing architectures, and loading conditions are required to evaluate the broader robustness of this criterion. From the engineering design perspective, selection of the fracture criterion should consider whether the notch geometry is predetermined or may vary during the design process. If the notch type is not specified or can be modified as part of structural design, the ASED-VIMC criterion appears to be the safest choice due to its lower sensitivity to notch geometry; of course, for the [0/90]_s_ printing architecture within the 1–4 mm range of notch root radius under symmetrical bending.

## Figures and Tables

**Table 1:** Mechanical properties of the MEX-printed ABS with symmetrical [0/90]s printing architecture [39].

Elastic Modulus, E (GPa)		Poisson’s Ratio, ν (-)		Ultimate Tensile Strength (MPa)	
1.16	(0.1)	0.34	(0.06)	36.6	(0.5)

**Table 2:** Process parameters of the MEX-printed ABS specimens.

Material	Height of Layer (mm)	Nozzle Diameter (mm)	Temperature of Nozzle (°C)	Temperature of Bed (°C)	Printing Speed (mm/s)	Width of Extrusion (mm)	Infill Percentage (%)
ABS	0.25	0.4	240	90	50	0.48	100

**Table 3:** Specifications of the flexural tests.

Lay-Up	Load Cell Capacity (kN)	Loading Rate (mm/min)	Loading Roller Diameter (mm)	Support Span (mm)
[0/90]s	5	10	5	96

**Table 4:** The experimentally acquired fracture loads (*P_EXi_*) of the pre-cracked and notched MEX-printed ABS specimens along with their corresponding average values (*P_av_*) and standard deviations (SD).

Type of Discontinuity	Nominal Notch Root Radius (mm)	Mean Actual Notch Root Radius (mm)	*P_Ex1_* (N)	*P_EX2_* (N)	*P_EX3_* (N)	*P_av_* (N)	*SD* (N)
U-notch	1	1.03	689	674	693	685	10.0
	2	2.08	630	634	607	624	14.6
	4	4.07	573	520	600	564	40.7
BV-notch	1	0.98	504	541	516	520	18.9
	2	2.00	504	521	554	526	25.4
	4	4.13	567	590	589	582	13.0
Pre-crack	0	0	562	607	581	583	22.6

**Table 5:** Critical parameters of the VIMC-based fracture criteria for the MEX-printed ABS notched specimens.

Printing Architecture	MS-VIMC Criterion	PS-VIMC Criterion	ASED-VIMC Criterion						
	$d_{c}$ (mm)	$r_{c}$ (mm)	$W_{c}$ (MJ/m^3^)	U-Notches			Blunt V-Notches		
				$R_{c}+\rho /2$ (mm)			$R_{c}+\rho /3$ (mm)		
				ρ = 1	ρ = 2	ρ = 4	ρ = 1	ρ = 2	ρ = 4
[0/90]_s_	4.74	1.19	0.58	2.86	3.36	4.36	2.69	3.02	3.69

**Table 6:** Experimental and theoretically-numerically predicted fracture loads of TPB cross-ply ABS specimens.

Type of Discontinuity	Nominal Notch Root Radius (mm)	Average Experimental Fracture Load (N)	PS-VIMC (N)	MS-VIMC (N)	ASED-VIMC (N)	XFEM-CZM-VIMC (N)
U-notch	1	685	587	558	635	509
	2	624	552	578	625	503
	4	564	545	608	600	533
BV-notch	1	520	594	617	566	489
	2	526	557	689	565	500
	4	582	546	688	561	523
Average discrepancy (%)			7.4	10.1	5.7	11.5

**Table 7:** Comparison of the average experimental results with theoretical-numerical predictions in terms of the percent difference.

	PS-VIMC (%)			MS-VIMC (%)			ASED-VIMC (%)			XFEM-CZM-VIMC (%)		
	ρ = 1	ρ = 2	ρ = 4	ρ = 1	ρ = 2	ρ = 4	ρ = 1	ρ = 2	ρ = 4	ρ = 1	ρ = 2	ρ = 4
U-notch	14.3	11.5	0.4	18.5	7.4	11.1	7.3	0.2	9.7	25.7	19.4	2.6
Blunt V-notch	9.0	8.0	1.4	5.9	8.7	8.8	6.0	7.4	3.6	6.0	4.9	10.1
Average (%)	7.4			10.1			5.7			11.5		

**Table 8:** Sensitivity of fracture-load predictions to the plane-stress and plane-strain formulations for representative notched specimens.

Criterion	Specimen	Plane-Stress Prediction (N)	Plane-Strain Prediction (N)	Differences (%)
XFEM-CZM-VIMC	U-notch (ρ = 4 mm)	533	532	0.2
XFEM-CZM-VIMC	BV-notch (ρ = 4 mm)	523	523	0.0
ASED-VIMC	U-notch (ρ = 4 mm)	600	569	5.1
ASED-VIMC	BV-notch (ρ = 4 mm)	561	539	3.9

(Note: Percentage differences are calculated relative to the plane-stress predictions).

## Data Availability

The raw data supporting the conclusions of this article will be made available by the authors on request.
